# Reconstructing the Genomic Content of Microbiome Taxa through Shotgun Metagenomic Deconvolution

**DOI:** 10.1371/journal.pcbi.1003292

**Published:** 2013-10-17

**Authors:** Rogan Carr, Shai S. Shen-Orr, Elhanan Borenstein

**Affiliations:** 1 Department of Genome Sciences, University of Washington, Seattle, Washington, United States of America; 2 Department of Immunology, Rappaport Institute of Medical Research, Faculty of Medicine and Faculty of Biology, Technion, Haifa, Israel; 3 Department of Computer Science and Engineering, University of Washington, Seattle, Washington, United States of America; 4 Santa Fe Institute, Santa Fe, New Mexico, United States of America; The Centre for Research and Technology, Hellas, Greece

## Abstract

Metagenomics has transformed our understanding of the microbial world, allowing researchers to bypass the need to isolate and culture individual taxa and to directly characterize both the taxonomic and gene compositions of environmental samples. However, associating the genes found in a metagenomic sample with the specific taxa of origin remains a critical challenge. Existing binning methods, based on nucleotide composition or alignment to reference genomes allow only a coarse-grained classification and rely heavily on the availability of sequenced genomes from closely related taxa. Here, we introduce a novel computational framework, integrating variation in gene abundances across multiple samples with taxonomic abundance data to deconvolve metagenomic samples into taxa-specific gene profiles and to reconstruct the genomic content of community members. This assembly-free method is not bounded by various factors limiting previously described methods of metagenomic binning or metagenomic assembly and represents a fundamentally different approach to metagenomic-based genome reconstruction. An implementation of this framework is available at http://elbo.gs.washington.edu/software.html. We first describe the mathematical foundations of our framework and discuss considerations for implementing its various components. We demonstrate the ability of this framework to accurately deconvolve a set of metagenomic samples and to recover the gene content of individual taxa using synthetic metagenomic samples. We specifically characterize determinants of prediction accuracy and examine the impact of annotation errors on the reconstructed genomes. We finally apply metagenomic deconvolution to samples from the Human Microbiome Project, successfully reconstructing genus-level genomic content of various microbial genera, based solely on variation in gene count. These reconstructed genera are shown to correctly capture genus-specific properties. With the accumulation of metagenomic data, this deconvolution framework provides an essential tool for characterizing microbial taxa never before seen, laying the foundation for addressing fundamental questions concerning the taxa comprising diverse microbial communities.

## Author Summary

Most microorganisms inhabit complex, diverse, and largely uncharacterized communities. Metagenomic technologies allow us to determine the taxonomic and gene compositions of these communities and to obtain insights into their function as a whole but usually do not enable the characterization of individual member taxa. Here, we introduce a novel computational framework for decomposing metagenomic community-level gene content data into taxa-specific gene profiles. Specifically, by analyzing the way taxonomic and gene abundances co-vary across a set of metagenomic samples, we are able to associate genes with their taxa of origin. We first demonstrate the ability of this approach to decompose metagenomes and to reconstruct the genomes of member taxa using simulated datasets. We further identify the factors that contribute to the accuracy of our method. We then apply our framework to samples from the human microbiome – the set of microorganisms that inhabit the human body – and show that it can be used to successfully reconstruct the typical genomes of various microbiome genera. Notably, our framework is based solely on variation in gene composition and does not rely on sequence composition signatures, assembly, or available reference genomes. It is therefore especially suited to studying the many microbial habitats yet to be extensively characterized.

## Introduction

Microbes are the most abundant and diverse life form on the planet. Recent advances in high-throughput sequencing and metagenomics have made it possible to study microbes in their natural environments and to characterize microbial communities in unprecedented detail. Such metagenomic techniques have been used to study communities inhabiting numerous environments, ranging from the bottom of the ocean [1], [2] and the roots of plants [3], [4] to the guts of mammals [5]. In particular, human-associated microbial communities have attracted tremendous attention, with several large-scale initiatives aiming to characterize the composition and variation of the human microbiome in health and disease [6]–[8]. Such studies have demonstrated a strong link between the microbiome and the health of the host, identifying marked compositional shifts in the microbiome that are associated with a variety of diseases [9]–[12].

Using a variety of experimental techniques and bioinformatic protocols [13], [14], metagenomics-based surveys can now characterize both the taxonomic and gene composition of the microbiome. Specifically, amplicon sequencing of conserved genes, such as the 16S ribosomal RNA gene, can be used to determine the relative abundance of each taxon [13], [15]. Obtained 16S sequences are clustered into Operational Taxonomic Units (OTUs), providing a proxy for the set of taxa found in the community [14], [16]. Alternatively, shotgun metagenomic sequencing can be used to derive short sequences (reads) directly from the community without amplification [17], [18]. These reads can then be mapped to a set of reference genes or orthologous groups (e.g., those defined by KEGG [19] or COG [20]) to translate read count data into relative abundances of functional elements, representing the collective set of genes found in the microbiome.

One of the key challenges in metagenomic research is the identification of the taxonomic origin for each shotgun metagenomic read or gene and, ultimately, the reconstruction of the genomes of member taxa directly from these reads. A diverse set of methods have been developed to parse shotgun metagenomic data and to obtain insights into the underlying taxa. These methods can be largely partitioned into several distinct categories, including: *alignment to reference genomes*, *taxonomic classification*, *assembly*, and *binning* (Figure S1). For ecosystems that are well-covered by reference genomes, such as the human microbiome [6], [21], alignment to reference genomes provides a way to determine the abundance of the various strains, species, or clades in the community [6], [8], [22] and can be used to assess strain variation within and between samples [23] (Figure S1A). Taxonomic classification methods, also referred to as taxonomic or phylogenetic binning, provide a less-specific phylogenetic label to each read, usually through a more permissive alignment to known sequences in nucleotide or peptide space [24], [25] (Figure S1B). These methods can be useful for determining the abundance of specific clades and for assisting with assembly efforts. These techniques, however, are limited by the set of reference genomes available and are only useful when relatively many community members have been previously sequenced. Considering the vast diversity of microbial communities and the challenges involved in isolation and culturing efforts [17], this approach can be applied on a large scale to only very few microbial communities.

When reference genomes are not available, assembly methods can be used to link reads into contigs and scaffolds that are easier to annotate [26] (Figure S1C). Such methods have been used to reconstruct full species [27], coexisting strains [28], [29], and more generally to construct catalogues of genes specific to particular or general ecosystems [6], [8], [12], [30]. Assembly is generally limited by the fraction of reads that can be mapped due to the complexity of most communities and the low coverage of each individual genome. Consequently, de-novo assembly of complete genomes from shotgun metagenomic samples is feasible only in extreme cases of low-complexity communities, very deep sequencing, or in combination with sample filtration techniques [27], [29], [31], [32]. Binning methods similarly aim to cluster reads into distinct groups, but do not necessarily require sequences to overlap (Figure S1D). Binning methods typically partition reads based on frequencies of nucleotide patterns (k-mers) [33]–[42], but can also use abundance and similarity metrics [12], [30], [41]–[43]. While these methods utilize every read in a metagenomic sample, they have several significant shortcomings. K-mer methods, for example, are constrained by the short length of each read, the low resolution of nucleotide usage profiling, assume homogeneity of coding bias both across genomes and locally, and may not accurately discriminate highly-related organisms. Furthermore, methods based on clustering do not usually allow reads, genes, or assemblies to be assigned to more than one group, which is problematic for highly conserved regions of a genome and for mapping reads from gene catalogs that use a low threshold on sequence identity [8].

Finally, in addition to the above well-established categories, yet another category of methods for parsing metagenomic data can be defined, which we refer to here as *deconvolution*. Deconvolution-based methods aim to determine the genomic element contributions of a set of taxa or groups to a metagenomic sample (Figure S1E). These methods profoundly differ from the binning methods described above as a single genomic element, such as a read, a contig, or a gene, can be assigned to multiple groups. An example of such a method is the non-negative matrix factorization (NMF) approach [44]–[46], a data discovery technique that determines the abundance and genomic element content of a sparse set of groups that can explain the genomic element abundances found in a set of metagenomic samples.

In this manuscript, we present a novel deconvolution framework for associating genomic elements found in shotgun metagenomic samples with their taxa of origin and for reconstructing the genomic content of the various taxa comprising the community. This *metagenomic deconvolution* framework (MetaDecon) is based on the simple observation that the abundance of each gene (or any other genomic element) in the community is a product of the abundances of the various member taxa in this community and their genomic contents. Given a large set of samples that vary in composition, it is therefore possible to formulate the expected relationships between gene and taxonomic compositions as a set of linear equations and to estimate the most likely genomic content of each taxa under these constraints. The metagenomic deconvolution framework is fundamentally different from existing binning and deconvolution methods since the number and identity of the groupings are determined based on taxonomic profile data, and the quantities calculated have a direct, physical interpretation. A comparison of the metagenomic deconvolution framework with existing binning and deconvolution methods can be found in Supporting Text S1.

We begin by introducing the mathematical basis for our framework and the context in which we demonstrate its use. We then use two simulated metagenomic datasets to explore the strengths and limitations of this framework on various synthetic data. The first dataset is generated with a simple error-free model of metagenomic sequencing that allows us to characterize the performances of our framework without the complications of sequencing and annotation error. The second dataset is generated using simulated metagenomic sequencing of model microbial communities composed of bacterial reference genomes and allows us to study specifically the effects of sequencing and annotation error on the accuracy of the framework's genome reconstructions. We finally apply the metagenomic deconvolution framework to analyze metagenomic samples from the Human Microbiome Project (HMP) [6] and demonstrate its practical application to environmental and host-associated microbial communities.

## Results

### The metagenomic deconvolution framework

Consider a microbial community composed of some set of microbial taxa. From a functional perspective, the genome of each taxon can be viewed as a simple collection of genomic elements, such as k-mers, genes, or operons. The metagenome of the community can accordingly be viewed as the union of these genomic elements, wherein the abundance of each element in the metagenome reflects the prevalence of this element in the various genomes and the relative abundance of each genome in the community. Specifically, if some genomic element is prevalent (or at least present) in a certain taxon, we may expect that the abundance of this element across multiple metagenomic samples will be correlated with the abundance of the taxon across the samples. If the abundances of both genomic elements and taxa are known, such correlations can be used to associate genomic elements with the various taxa composing the microbial community [47], [48]. In Supporting Text S1, we evaluate the use of a simple correlation-based heuristic for predicting the genomic content of microbiome taxa and find that such simple correlation-based associations are limited in accuracy and are extremely sensitive to parameter selection. This limited utility is mostly due to the fact that associations between genomic elements and taxa are made for each taxon independently of other taxa, even though multiple taxa can encode each genomic element and may contribute to the overall abundance of each element in the various samples. We therefore present here a statistical deconvolution framework, improving upon the simple correlation metric and developing a mathematical model of shotgun metagenomic sequencing. This model quantifies the associations between a genomic element found in a set of samples and all the taxa in the community simultaneously, providing an estimate for the prevalence of this element in the genome of each taxon. Such statistical approaches have proven successful in analyzing gene expression data, allowing, for example, to deconvolve microarray data from mixed tissue samples into cell type-specific expression profiles [49].

Formally, if 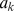 denotes the abundance of genome *k* in the community and 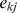 denotes the prevalence of an element *j* in genome *k* (e.g., in terms of copy number or length in nucleotides), the total abundance of this element in the community can be represented as:
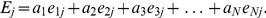
(1)Note that similar models have been used as the basis for simulating shotgun metagenomic sequencing [50]–[53], and the total abundance of the element in the community is independent of the individual genome sizes. Now, assume that the total abundances of genomic elements, 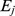, can be determined through shotgun metagenomic sequencing, and that the abundances of the various genomes, 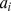, can be obtained using 16S sequencing or from marker genes in the shotgun metagenomic data [54], [55]. Accordingly, in Eq. (1) above, the only terms that are unknown are the prevalence of each genomic element in each genome, 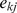, and these are the specific quantities required to functionally characterize each taxon in the community.

Clearly, if only one metagenomic sample is available, Eq. (1) cannot be used to calculate the prevalence of the genomic elements 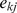. However, assume *M* different metagenomic samples have been obtained, each representing a microbial community with a somewhat different taxonomic composition. For each genomic element, we can now write a system of linear equations of the form:
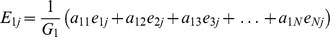


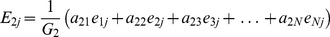
(2)…
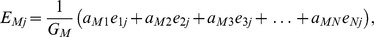
or more compactly as
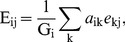
(3)where the subscript *i* denotes the sample and 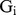 is a normalization coefficient (see below). Given enough samples, 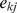, the prevalence of a given genomic element *j* in each taxon, can be analytically solved by linear regression. Repeating this process for all genomic elements found in the community, we can therefore obtain an estimate of the prevalence of each element (e.g. each gene) in each taxon, effectively reconstructing the genomic content of all community members.

The normalization constant 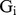 represents, technically, the total amount of genomic material in the community. Clearly, 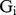 is not known *a priori* and in most cases cannot be measured directly. Assume, however, that some genomic element is known to be present with relatively consistent prevalence across all taxa in the community. Such an element can represent, for example, certain ribosomal genes that have nearly identical abundances in every sequenced bacterial and archaeal genome (see Methods). We can then rewrite Eq. (3) in terms of this constant genomic element, 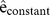 with a total abundance in sample *i*, 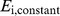:
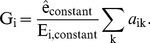
(4)Assuming that the taxonomic abundances have been normalized to sum to 1, this simplifies to
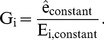
(5)We can accordingly substitute 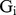 in Eq. (3) with this term, obtaining a simple set of linear equations where the only unknown terms are the prevalence of each genomic element in each taxon, 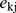.

### Implementation of the metagenomic deconvolution framework

Metagenomic deconvolution is a general framework for calculating taxa-specific information from metagenomic data. Notably, this framework is modular, comprising four distinct components: (i) determination of taxonomic composition in each sample, 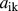; (ii) determination of the abundances of genomic elements in each sample, 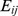; (iii) selection of a constant genomic element, 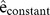; and (iv) calculation of the taxa-specific genomic element abundances, 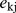, by solving Eq. (3). Each of these components can be implemented in various ways. For example, different metagenomic techniques, sequence mapping methods, and annotation pipelines can be used to determine the abundance of various genomic elements in each sample. Genomic elements can represent k-mers, motifs, genes, or other elements that can be measured in the samples and whose taxonomic origin are unknown. Similarly, there are multiple regression methods that can be applied to solve the set of equations obtained and to estimate 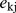, including least squares regression, non-negative least squares regression, and least squares regression with L1-regularization (e.g., lasso [56]). Finally, the taxonomic abundances need not be derived necessarily from 16S sequencing but can rather be determined directly from metagenomic samples [54], [55].

In this study, we used gene orthology groups (which we will mostly refer to simply as genes), specifically KEGG orthology groups (KOs) [19], as the genomic elements of interest in Eq. (3) above. In this context, we defined the abundance of a KO in a metagenomic sample, 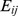, as the number of reads mapped to this KO, and the prevalence of a KO in a genome, 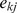, as the number of nucleotides encoding it in the genome. We accordingly applied our deconvolution framework to predict the length of each KO in each genome, ultimately obtaining ‘reconstructed’ genomes in the form of a list of all the KOs present in a genome and their predicted lengths.

We used the 16S rRNA gene as the constant genomic element, 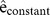, to calculate the normalization coefficient 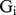. The length of the 16S gene is largely consistent across all sequenced archaeal and bacterial strains. When the abundances of the 16S gene across shotgun metagenomic samples, 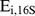, are not available, other genes or groups of genes with a consistent length across the various taxa can also be used. Specifically, in applying our framework to metagenomic samples from the HMP below, we used a set of bacterial and archaeal ribosomal genes to estimate 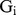 (Methods). Finally, we used least squares and non-negative least squares regression to solve Eq. (3) and to estimate 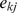 (Methods). Notably, such regression techniques require that there are at least as many samples as taxa in order for there to be a solution. However, if there are fewer samples than taxa, regularized regression techniques, such as the lasso [56], can be used. For each dataset presented in this manuscript, we have evaluated the solutions presented by these regression methods and compared their accuracies across the different datasets in Supporting Text S1.

Notably, in many cases, our key goal is to determine which genes are present in (or absent from) a given genome, rather than their exact length (e.g., in nucleotides) in this genome. To predict the presence or absence of a gene in a genome, we used a simple threshold-based method. Specifically, we compared the predicted length of each gene to the average length of this gene across sequenced genomes. Genes for which the ratio between these two values exceeded a certain threshold were predicted to be present. For example, we could predict that a gene is present in a genome if it is predicted to have a length greater than 0.5 the average length of all sequenced orthologs of this gene. This method will also allow us to correct for inaccuracies in length predictions. In the results reported below, we further demonstrate the robustness of reconstructed genomes to threshold value selection.

### Deconvolving simple synthetic metagenomic samples

We first use a simple model of metagenomic sampling to characterize metagenomic deconvolution in the absence of sequencing and annotation errors. To this end, we simulated microbial communities composed of 60 “species” of varying abundances (see Methods). In this model, each species was defined as a collection of “genes” assigned randomly from a total set of 100 gene orthology groups. These genes had no sequence, were assumed to vary in length, and could be present in multiple copies in each genome. 100 model microbial communities were generated with different, but correlated, abundances for each member species (Figure S2). The relative abundances of each species in the communities were assumed to be known (e.g. from targeted 16S sequencing). Metagenomic samples consisting of 5M reads were generated, simulating shotgun sequencing through a random sampling process weighted by the relative abundance of each gene in the community. Reads were assumed to map without error to the appropriate orthology group, counting towards the observed relative abundance of each gene orthology group in the sample. Full details of the model are given in the Methods.

**Figure 1:**
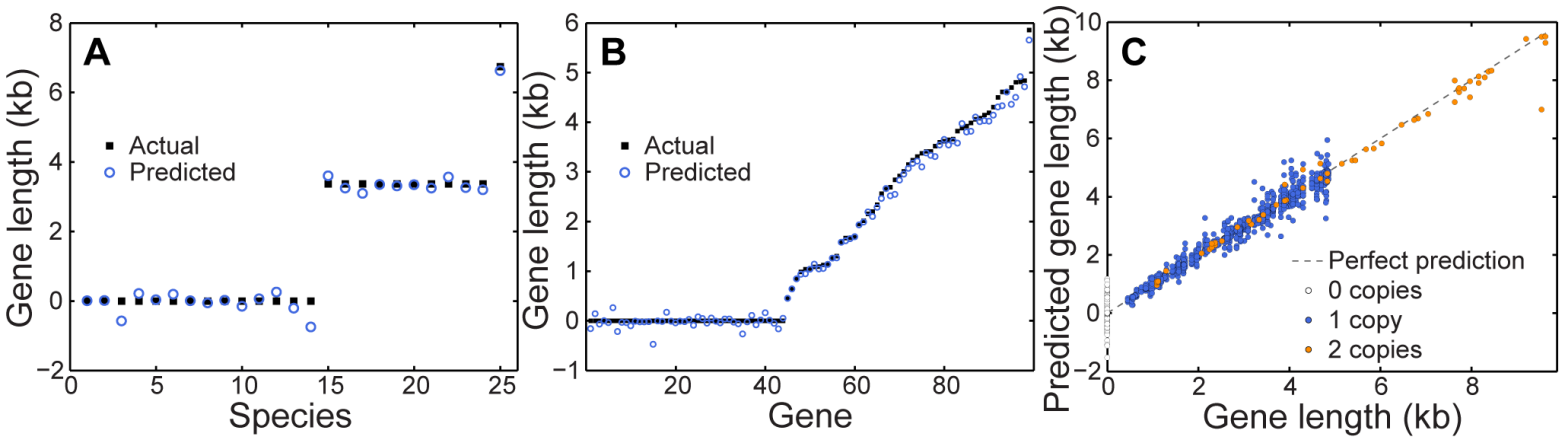
Metagenomic deconvolution successfully predicted the length of each gene in the various species found in simple synthetic metagenomic samples (see text). Actual (black squares) and predicted (blue circles) gene lengths for a given gene in each species (**A**) and for all the genes in one species (**B**). The specific gene and the specific species shown here were those with the median variation in abundance across samples. (**C**) The predicted gene length as a function of the actual length for all genes in all species. Different colors are used to indicate the number of copies in a species. The dashed line represents a perfect prediction. Note that predicted gene lengths can be negative, as predictions were made in this case using least-squares regression. Gene lengths can be restricted to positive values using alternative regression methods (see Supporting Text S1).

We applied the deconvolution framework described above to predict the length of each gene in each species. Examining the predicted length of a typical gene across all species, we found that we successfully predicted the actual genomic length of this gene among the different species (Figure 1A). Similarly, comparing the predicted lengths of all genes in a typical species to the species' actual genome, we find that our framework accurately reconstructed the genomic content of the species, successfully identifying absent genes and correctly estimating a wide range of gene lengths (Figure 1B). Furthermore, analysis of the predictions obtained for all genes and for all species in the community clearly demonstrates that the metagenomic deconvolution framework can effectively reconstruct gene lengths across all genomes, orthology groups, and copy numbers (Figure 1C).

Clearly, the predicted gene lengths described above, while accurate, are not perfect, and may be affected by various sources of noise in the data. Moreover, as noted above, in many cases, we are primarily interested in predicting whether a gene is present in a certain genome rather than in determining its exact length. Converting the predicted gene lengths to gene presence/absence predictions using a threshold of 0.5 of the gene length, we find that we are able to correctly predict the presence and absence of all genes in all species with 100% accuracy. We further confirmed that this result is robust to the specific threshold used, with all thresholds values between 0.2 and 0.8 yielding perfect predictions (Figure S3).

### Determinants of prediction accuracy

**Figure 2:**
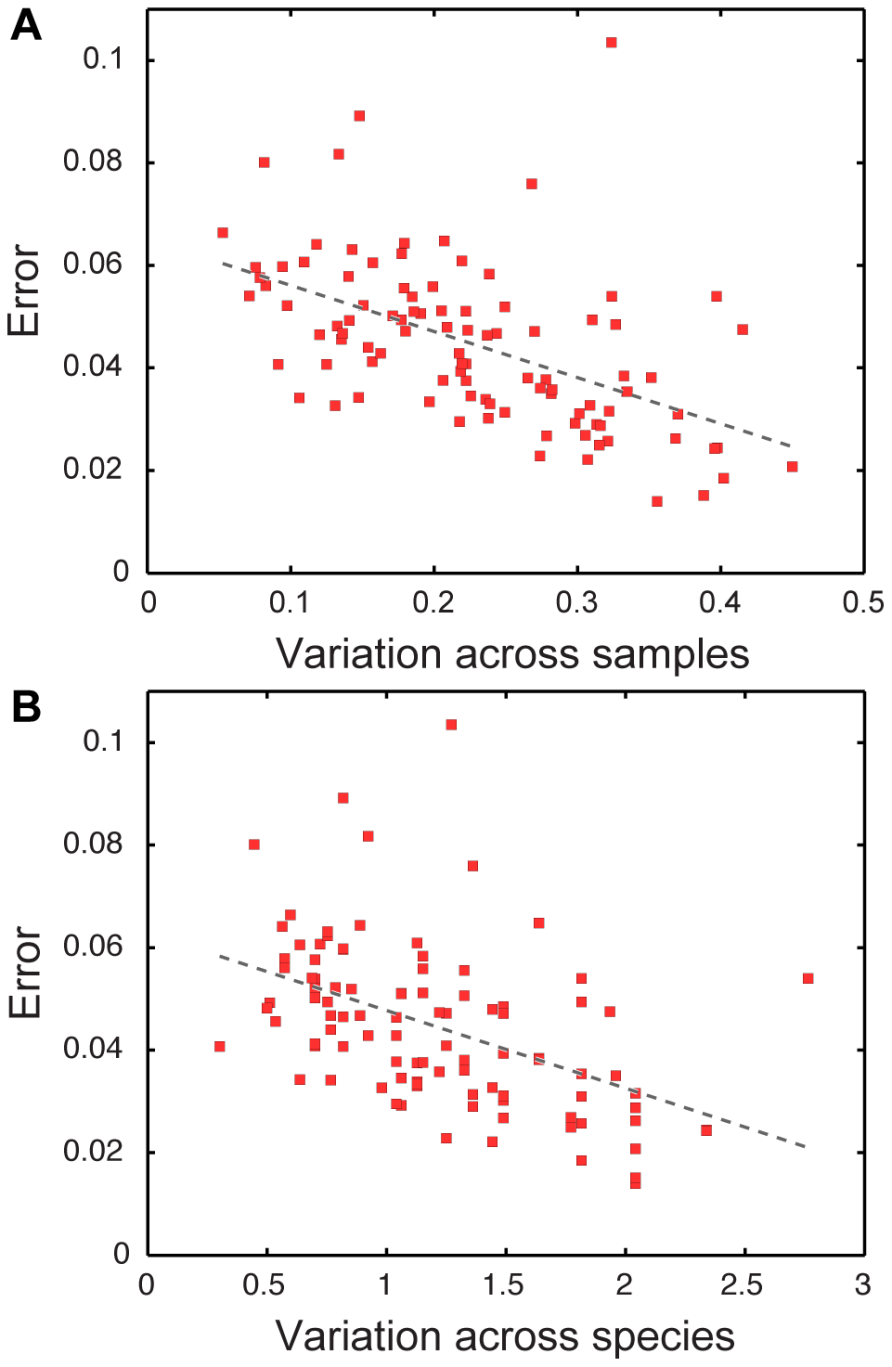
Prediction accuracy is correlated with variation. Average error in prediction accuracy for each gene orthology group (red squares) as a function of the variation (standard deviation divided by the mean) across samples, R = −0.48, p<4.3×10^−7^ (**A**), and across species, R = −0.53, p<2.0×10^−8^ (**B**). Best fit lines are illustrated. Error is calculated as the relative error in the length prediction for each gene orthology group.

Predictions of a given gene's length across the species vary in accuracy from gene to gene, with some genes having a noticeably higher overall error than others (Figure 1C). By examining the distribution of genes among samples and species, we find that prediction accuracy for a gene is significantly correlated with its level of variation across samples (Figure 2A) and across species (Figure 2B), with more variable genes having lower prediction error on average. These patterns in prediction accuracy are not surprising. Since our framework is based on detecting species and gene abundances that co-vary, highly variable genes or species carry a stronger signal and lead to more accurate predictions.

Interestingly, however, this seemingly limiting link between prediction accuracy and variation is one of the strengths of our framework, as it provides better accuracy for predicting exactly the genes that are of most interest. Specifically, genes that vary from species to species are those that confer species-specific functional capacity and are those that are most crucial for characterizing novel genomes. Similarly, genes that vary most from sample to sample are those endowing each community with specific metabolic potential and are therefore often of clinical interest. In contrast, genes with little variation from species to species and from sample to sample are likely to include many housekeeping genes, whose presence in each genome is not surprising and can mostly be assumed *a priori*.

Clearly, many microbial communities exhibit high species diversity and are inhabited by an extremely large number of species, challenging deconvolution efforts. Moreover, the abundances of species across samples are not independent: In a given environment, some species may dominate all samples, while other species may tend to be rare across all samples. Interactions between species may also introduce correlations between the abundances of various species. These inter-sample and inter-species correlations might also affect our ability to correctly deconvolve each member species, as they in effect reduce the level of variation in the data. For example, species with highly correlated abundances (e.g., the set of dominant species across all samples) will contribute similarly to the abundances of genes in the various samples and will be hard to discriminate. To explore the impact of the number of species in the community and of correlations between species abundances on metagenomic deconvolution, we used an additional set of simulated communities. Specifically, metagenomic samples were generated with a varying number of species and a varying level of inter-sample correlation in species abundances (Methods; Figure S4). We find, as expected, that the accuracy (Figure S5A) and recall (Figure S5B) of deconvolution decreases as the number of species increases (assuming a constant sampling depth). Furthermore, increasing the level of correlation between species abundances across samples similarly results in reduced accuracy and recall (Figure S5).

### Deconvolving synthetic microbial communities with sequencing and annotation errors

The simple model presented above allowed us to explore the metagenomic deconvolution framework in ideal settings where reads are assumed to be error free and to unambiguously map to genes. We next set out to examine the application of our framework to synthetic metagenomic samples that incorporate both next-generation sequencing error and a typical metagenomic functional annotation pipeline. To this end, we simulated metagenomic sampling of microbial communities composed of three reference genomes (Methods). We specifically focused on strains that represent the most abundant phyla in the human gut, as determined by the MetaHIT project [8], and for which full genome sequences were available. Furthermore, these strains represented different levels of coverage by the KEGG database (which we used for annotation), ranging from a strain for which another strain of the same species exists in the database, to a strain with no member of the same genus in the database (Methods).

Ten communities with random relative strain abundances were simulated. The relative abundances in each community were assumed be to known through targeted 16S sequencing. For the analysis below, relative abundances ranged over a thousand-fold, but using markedly different relative abundance ratios had little effect on the results (see Supporting Text S1). Shotgun metagenomic sequencing was simulated using Metasim [50], with 1M 80-base reads for each sample and an Illumina sequencing error model (Methods). The abundances of genes in each metagenomic sample were then determined using an annotation pipeline modeled after the HMP protocol [47], with reads annotated through a translated BLAST search against the KEGG database [19]. To assess the accuracy of this annotation process and its potential impact on downstream deconvolution analysis, we first compared the obtained annotations to the actual genes from which reads were derived. Overall, obtained annotation counts were strongly correlated with expected counts (0.83, *P*<10^−324^; Pearson correlation test; Figure S6). Of the reads that were annotated with a KO, 82% were annotated correctly. Notably, however, only 62% of the reads originating from genes associated with KOs were correctly identified and consequently the read count for most KOs was attenuated. Highly conserved genes, such as the 16S rRNA gene, were easily recognized and had relatively accurate read count (Figure S6). Full details of this synthetic community model and of the sequencing simulations are provided in Methods.

**Figure 3:**
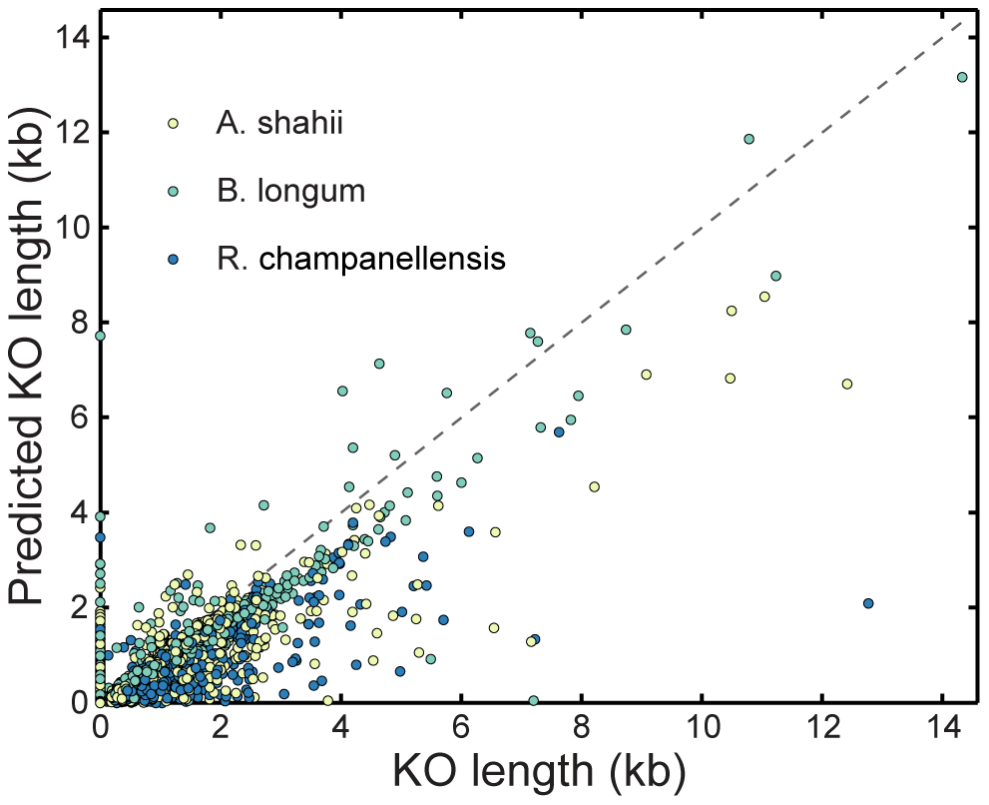
Predicting the length of each KO in each species using deconvolution and the effect of annotation errors. Predicted KO lengths vs. actual KO lengths, using BLAST-based annotation.

We deconvolved each KO using the obtained abundances to predict the length of each KO in each genome. We found that the predicted lengths were strongly correlated with the actual lengths (rho 0.84, *P*<10^−324^; Pearson correlation test), although for most KOs predicted lengths were shorter than expected (Figure 3). This under-prediction of KO lengths can be attributed to the normalization process. Specifically, as noted above, the detected abundances of conserved genes used for normalization tended to be less attenuated by the annotation pipeline than the abundances of other genes, which were therefore computed to be shorter than they actually were. Notably, some KOs that are in fact entirely absent from the genomes under study were erroneously detected by the annotation pipeline and consequently predicted to have non-negligible lengths in the reconstructed genomes (Figure 3). To discriminate the error stemming from the annotation pipeline from error stemming directly from the deconvolution process, we reanalyzed the data assuming that each read was correctly annotated. We found that with the correct annotations, predicted KO lengths accurately reflected the actual length of each KO in each genome (rho 0.997, *P*<10^−324^; Pearson correlation test; Figure S7).

**Figure 4:**
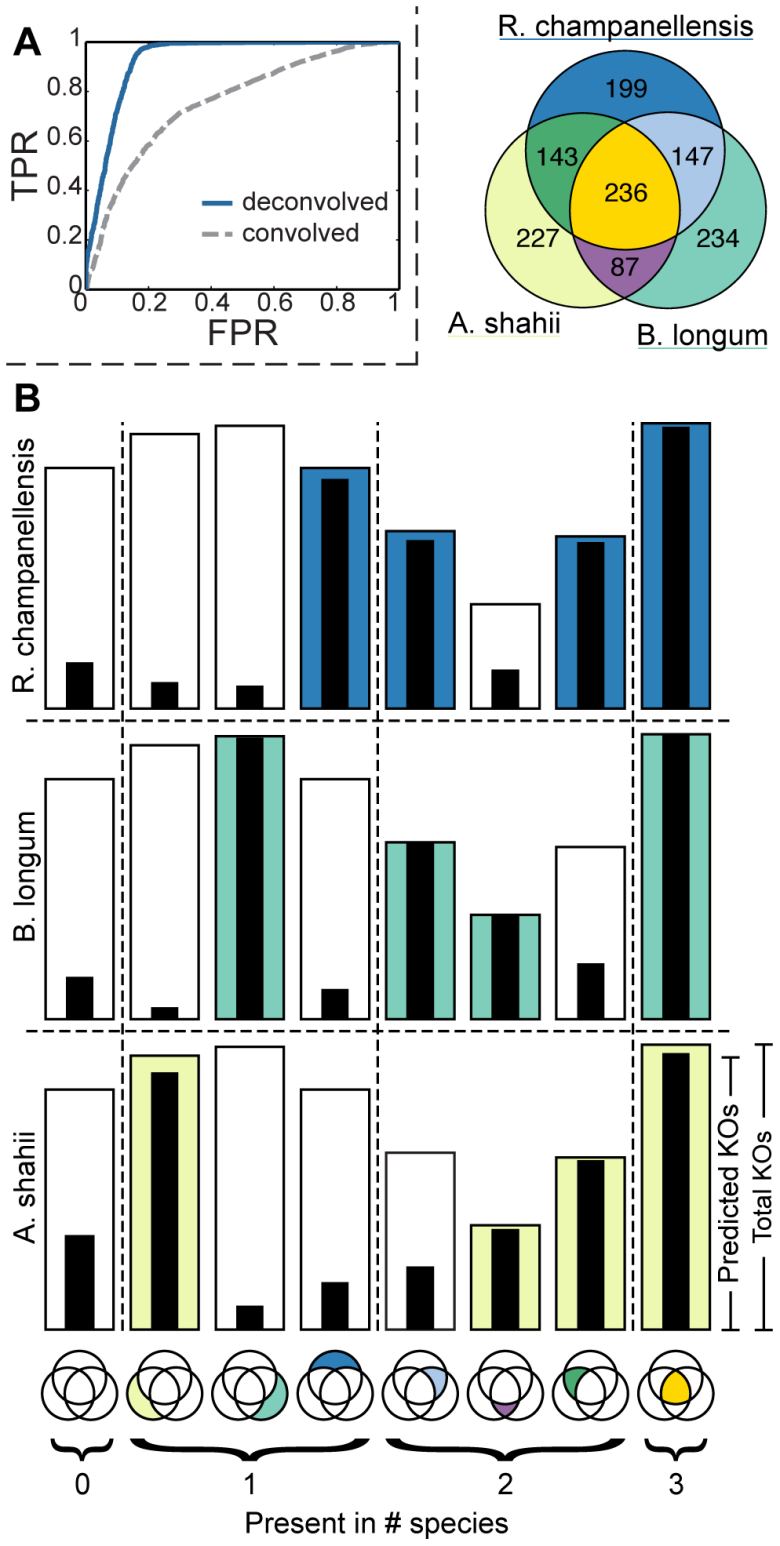
Reconstructing the genomic content of reference genomes from simulated mixed metagenomic samples using metagenomic deconvolution. (**A**) ROC curves (AUC = 0.93) for predicting KO presence and absence across all species as a function of the threshold used to predict the presence of a KO. ROC curve for a naïve convolved prediction (AUC = 0.76) is illustrated for comparison. (**B**) Predicted genomic content of each species. KOs are partitioned into bins based on the set of genomes in which they are present (e.g., genes present only in the first species, genes present only in the second species, genes present in the first and second species but not in the third, etc.; see Venn diagram). The height of each bar represents the proportion of KOs in each bin and the color represents the presence of these KO in each species. The black strip inside each bar represents the fraction of KOs from this bin predicted to be present in each species.

Importantly, while the error introduced by the annotation pipeline significantly affects the accuracy of predicted KO lengths, the presence (or absence) of each KO in each genome can still be successfully predicted by the threshold approach described above (Figure 4A). Specifically, using a threshold of 0.1 of the average length of each KO, metagenomic deconvolution reached an accuracy of 89% (correctly predicting both KO presence and absence) and a recall of 98% across the various genomes. Figure 4B further illustrates the actual and predicted genomic content of each strain, demonstrating that the method can accurately predict the presence of the same KO in multiple strains, highlighting the difference between the metagenomic deconvolution framework and existing binning methods (see also Discussion). We compared these predictions to a naïve ‘convoluted’ prediction (see Methods), confirming that deconvolution-based predictions were significantly more accurate than such a convoluted null model regardless of the threshold used (*P*<10^−324^, bootstrap; Figure 4A). For example, using a threshold of 0.1 as above, convoluted genomes were only 54% accurate. Considering the determinants of prediction accuracy described above, we further confirmed that prediction accuracy markedly increased for highly variable and taxa-specific genes (Supporting Text S1).

Given the noisy annotation process, we again set out to quantify the contribution of annotation inaccuracies to erroneous presence/absence predictions in the reconstructed genomes. As demonstrated in Figure 4B, most KO prediction errors were false positives – KOs wrongly predicted to be present in a strain from which they were in fact absent. Examining such KOs and the annotation of reads in each genome, we found that 99% of the false positive KOs were associated with mis-annotated reads, suggesting that deconvolution inaccuracies in these settings could be attributed almost entirely to erroneous annotation rather than to the deconvolution process itself. We again confirmed that when correct annotations are assumed, both accuracy and recall increase to more than 99%.

The analysis above was used to evaluate the impact of sequencing and annotation error on the metagenomic deconvolution framework using simulated metagenomic datasets generated from simple 3-strain communities. In Supporting Text S1, we further present a similar analysis, using simulated metagenomic samples generated from 20-strain communities and based on the HMP Mock Communities. We show that our framework obtains similar reconstruction accuracies for these more complex communities (Figure S8).

### Application of the deconvolution framework to metagenomic samples from the Human Microbiome Project

Finally, we considered human-associated metagenomic samples to demonstrate the application of the metagenomic deconvolution framework to real metagenomic data from highly complex microbial communities. These datasets further represent an opportunity to evaluate genome reconstructions obtained by our framework owing to the high-coverage of the human microbiome by reference genomes [6], [21] that can be used for evaluation. The Human Microbiome Project [6], [14] has recently released a collection of targeted 16S and shotgun metagenomic samples from 242 individuals taken from 18 different body sites in an effort to comprehensively characterize the healthy human microbiome. These human-associated microbial communities are diverse, with several hundred to several thousand 16S-based OTUs (operational taxonomical units clustered at 97% similarity) per sample and a total of more than 45,000 unique OTUs across all HMP samples. These OTUs represent bacteria and archaea from across the tree of life, including many novel taxa [57], and their diversity is in agreement with shotgun metagenomics-based measures [6]. Clearly, the high number of unique OTUs in each sample does not permit deconvolution and genome reconstruction at the OTU level. Moreover, these OTUs do not represent individual species, but rather distinct sequences accurate to only a genus-level phylogenetic classification [6]. Examining the phylogenetic distribution of the taxa comprising the microbiome suggests that certain body sites, such as the tongue dorsum, are dominated by relatively few genera. This allows us to use metagenomic deconvolution at the genus level, predicting the most likely genomic content of the various genera found in the microbiome. Reconstructed genus-level genomes can be viewed as the average genomic content across all present strains in the genus, providing insight into the capacities of the various genera. Moreover, while many species inhabiting the human microbiome have not yet been characterized or sequenced, most human-associated genera include at least a few fully sequenced genomes, allowing us to assess the success of our framework and the accuracy at which reconstructed genera capture known genus-level properties. Notably, however, microbial communities from other environments or from other mammalian hosts often harbor many uncharacterized taxa, even at levels higher than genera [58], [59], making a genus-level deconvolution a still biologically relevant goal.

We accordingly applied our deconvolution framework to HMP tongue dorsum metagenomic samples (Methods). OTU abundances and taxonomic classification were obtained from the HMP QIIME 16S pipeline [14]. KO abundances were obtained from the HMP HUMAnN shotgun pipeline [19]. In total, 97 tongue dorsum samples had both OTU and KO data available. OTUs were pooled to calculate the relative abundance of each genus in each sample. After pooling, we identified 14 genera that dominated the tongue dorsum. We deconvolved these samples to obtain reconstructed genera and computed KO presence/absence in each reconstructed genus using a threshold of 0.25 copies.

**Figure 5:**
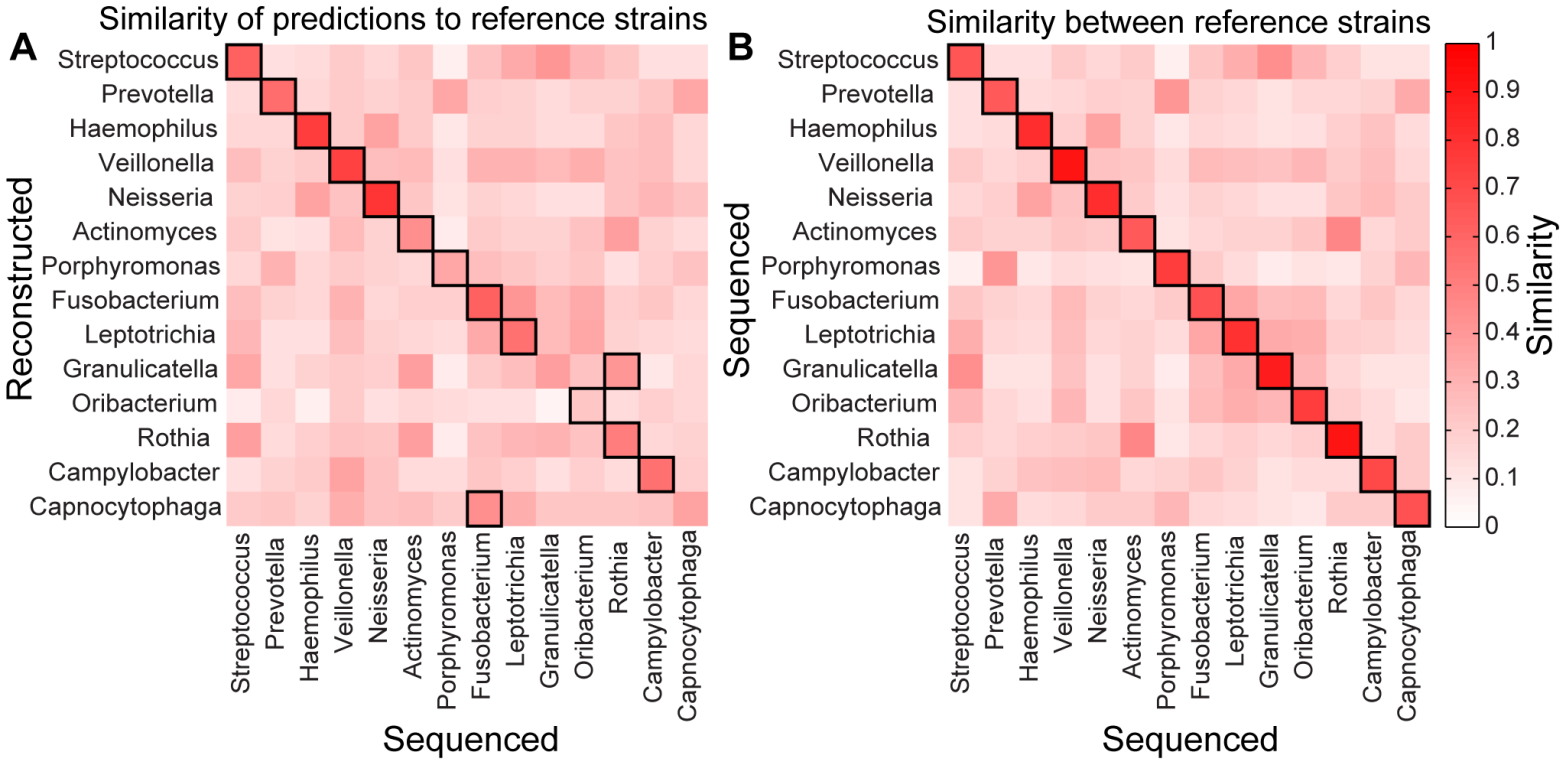
Reconstructing the genomic content of genera from HMP tongue dorsum samples. (**A**) The average similarity in KO content between each reconstructed genus and sequenced genomes from the various genera. Similarity is measured by the Jaccard similarity coefficient, over the set of the 500 KO with the highest variation across samples. Genera are ordered by their mean abundance in the set of samples under study. Entries highlighted with a black border represent the highest similarity in each row. (**B**) The average similarity between sequenced genomes from the various genera. Similarity was measured as in panel A.

To evaluate our predictions, we calculated the similarity between the 14 reconstructed genera and every sequenced genome from these genera (Methods). We find that 12 of the 14 reconstructed genera are most similar to genomes from the correct genus (Figure 5A). Interestingly, Capnocytophaga, one of the two reconstructed genera that did not most closely resemble genomes from its own genus, was the least abundant genus and appeared to be most similar to genomes from the Fusobacterium genus, with which it significantly co-occurs in the tongue dorsum [60]. This potentially reflects the sensitivity of deconvolution to highly correlated taxonomic abundances (see Discussion). Furthermore, overall, the observed similarities between each reconstructed genus and sequenced genomes from other genera (Figure 5A) largely reflect inter-genus similarities between the genomes from the various genera (Figure 5B). For example, although the reconstructed Prevotella is most similar to sequenced genomes from the Prevotella genus, it also exhibits high similarity to genomes from Porphyromonas and Capnocytophaga, two other genera from the Bacteroidetes phylum with relatively similar genomic content. These findings suggest that our deconvolution framework was able to accurately capture the similarities and the differences between the various genera based solely on variation in KO and OTU abundances across samples.

**Figure 6:**
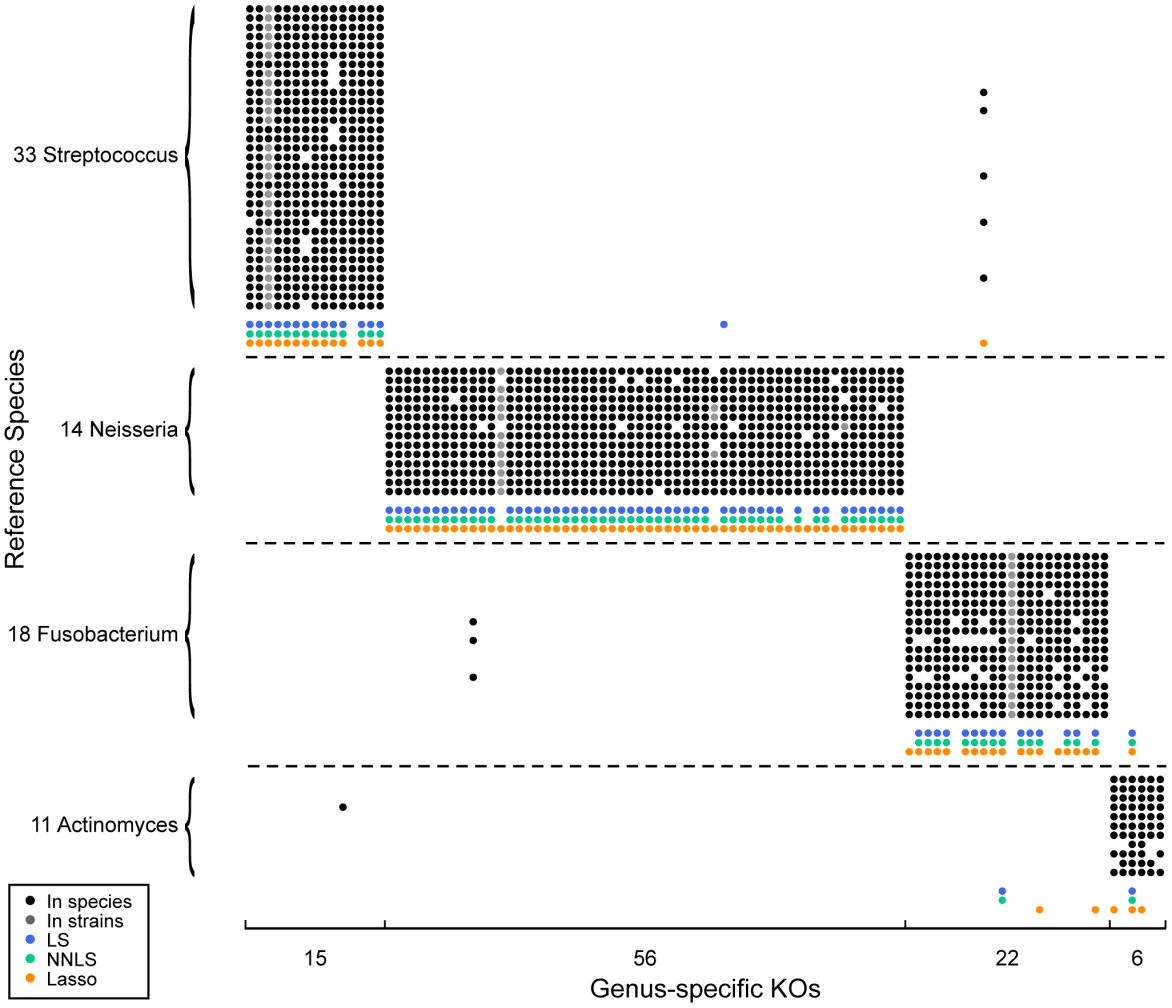
Predicting genus-specific KOs in genera from the HMP tongue dorsum samples. To restrict our analysis to well-sampled genera, only genera for which at least 10 reference genomes are available and for which at least 5 genus-specific KOs were obtained are considered. The presence (or absence) of genus-specific KOs across the set of sequenced species from each genus is illustrated by the presence (or absence) of a black dot. Gray dots indicate that the KO was present in only a subset of the sequenced strains of that species. KOs predicted to be present by metagenomic deconvolution are shown using colored dots. Results are shown for several regression methods, including least squares (LS, 89% accuracy, 82% recall), non-negative least squares (NNLS, 90% accuracy, 82% recall), and lasso (90% accuracy, 92% recall). See also Supporting Text S1.

To further study the capacity of genus-level deconvolution to reconstruct and characterize the various genera in the microbiome, we next focused on the set of genes that best distinguish one genus from the other. Clearly, even within a genus, the set of genes present in a genome varies greatly from species to species and from strain to strain. Yet, for each genus, a small number of genes that are present in almost every genome from that genus and that are absent from most other genomes can be found. These *genus-specific* genes best typify the genus, potentially encoding unique genus-specific capacities. Moreover, since such genes are consistently present or consistently absent within each genus, genus-level deconvolution is not complicated by the genus-level pooling of genomes. We defined genus-specific KOs as those present in 80% of the genomes from a given genus and in less than 20% of all others HMP reference genomes. We found in total 99 such KOs across 4 genera. Examining the reconstructed genera, we found that our framework successfully predicted the presence or absence of these genus-specific KOs (90% accuracy and 82% recall; Figure 6). Increasing the stringency for our definition and focusing on the 63 KOs that appeared in 90% of the genomes from a certain genus and in less than 10% of all others genomes further increased the accuracy (92%) and recall (94%) of our reconstructed genera. Predictions obtained using alternative regression methods were similarly accurate (see Supporting Text S1; Figures 6, S9).

### Comparison with binning and deconvolution methods

The metagenomic deconvolution framework introduced in this manuscript is a technique for associating genomic elements found in shotgun metagenomic samples with their taxa of origin and for reconstructing the genomic content of the various taxa comprising the community. Many different approaches have been developed to create such groupings of metagenomic features. Broadly, these methods fall into one of two categories, “binning” or “deconvolution”, depending on whether the genomic elements can be assigned to more than one group or not. As demonstrated in Supporting Text S1 (and see also Table S2), the differences between the metagenomic deconvolution framework and these existing methods originate primarily from the different mathematical frameworks employed by the various methods.

Binning methods, such as metagenomic linkage analysis [12], metagenomic clustering analysis [30], and MetaBin [43], are designed to cluster genomic elements that can only exist in one taxon (or group). Specifically, metagenomic linkage analysis clusters genes into groups based on their abundances and phylogeny across sets of metagenomic samples using the CHAMELEON algorithm [61]. Similarly, metagenomic clustering analysis clusters genes into groups based on their abundances across sets of metagenomic samples using the Markov clustering algorithm [62]. MetaBin, on the other hand, clusters individual reads based on their sequence similarities and abundances across sets of metagenomic samples using k-medoids clustering. As these methods all cluster genomic elements into distinct groups, they cannot correctly distribute elements that exist in multiple taxa (or groups), making them less appropriate for addressing questions of core vs. shared genome content (and see, for example, refs [54], [63], [64]). As we demonstrate in Supporting Text S1, these methods accordingly could not be used to reconstruct the genomic content of the three strains present in the simulated metagenomic samples incorporating sequencing and annotation error in terms of the gene orthology groups identified in the samples.

In contrast, deconvolution methods, such as non-negative matrix factorization (NMF) [44]–[46] and the proposed metagenomic deconvolution framework, are designed to assign genomic elements to multiple taxa. Specifically, NMF is a data discovery and compressed sensing tool that is designed to create a set number of groupings of elements that best fits the observed samples by factoring the feature matrix (here, the genomic elements found across a set of metagenomic samples) into two matrices. One matrix represents the abundance of the set of groups in each sample, and the other represents the distribution of genomic elements among these groups. The optimal number of groups can be determined from the fit of the matrix factorization to the original matrix [45], [46] or the stability of the solutions for a given number of groups [44]. Importantly, while NMF utilizes a mathematically similar approach to the metagenomic deconvolution framework, and can thus theoretically obtain comparable accuracies (see also Supporting Text S1), the two represent fundamentally different techniques. First, the groups identified by NMF are unlabeled, while those used by the metagenomic deconvolution framework by definition have a distinct taxonomic identity. Furthermore, the optimal number of groups detected in a set of samples by NMF does not necessarily correspond to any phylogenetic groupings present in the set of samples. Indeed, NMF does not group the gene orthology groups present in the simulated metagenomic samples incorporating sequencing and annotation error into strain-specific groupings (Supporting Text S1). Second, in the metagenomic deconvolution framework, the calculated quantities of genomic elements in each group have a direct physical interpretation (i.e. gene length or copy number), while NMF calculates coefficients without assigning a clearly interpretable meaning. Lastly, NMF functions on the entire set of genomic elements present in a set of samples (the feature matrix) as a whole, whereas the metagenomic deconvolution framework solves for the distribution of each genomic element among the various groups independently. This separability allows for custom regression techniques to be used for each genomic element (for example, regularized regression like lasso can be used for those genomic elements that are sparsely distributed) and the option to target only those genomic elements of interest.

## Discussion

In this study, we presented a novel framework for deconvolving shotgun metagenomic samples and for reconstructing the genomic content of the member microbial taxa. This metagenomic deconvolution framework utilizes the magnitude by which abundances of taxa and of genomic elements co-vary across a set of metagenomic samples to identify the most likely genomic content of each taxon. Above, we have described the mathematical formulation of this framework, detailed computational considerations for implementing it, characterized its performance and properties on synthetic metagenomic datasets, and demonstrated its practical use on metagenomic samples from the Human Microbiome Project.

The metagenomic deconvolution framework represents a fundamentally different approach to associating genomic elements found in shotgun metagenomic samples with the taxa present than the approaches employed by previously introduced methods. For example, methods relying on alignment to reference genomes [6], [8], [22], [24], [25] are heavily dependent on the availability of sequenced genomes from community members or from closely related species. As metagenomics research expands and researchers set out to characterize new environments inhabited by many novel, diverse, and never before seen species, such methods may be challenged by the scarcity of reference genomes and by the low phylogenetic coverage of many genera across genomic databases. In contrast, our method does not require reference genomes (see also below). Moreover, metagenomic deconvolution uses a mathematical model of shotgun sequencing to directly calculate the desired quantities of genomic elements (such as gene lengths or copy numbers) in specific taxa (such as a strain or genus), rather than to create groupings of elements that best fit the measured distribution.

Metagenomic deconvolution associates genomic elements with genomes of present taxa by identifying genomic elements that co-vary in abundance with organisms. As demonstrated above, this approach brings about an important advantage: The more variation of a given genomic element across samples and organisms, the more accurately it will be assigned to the various taxa. The deconvolution framework can accordingly be thought to be tuned to best identify those elements that make a taxon or a set of samples unique and that are therefore of most biological interest. Moreover, to a large extent, in analyzing the way gene and taxonomic abundances co-vary across the set of samples under study, it utilizes orthogonal, self-constrained information. Notably, the specific implementation presented in this study utilizes functional read annotation and therefore required a set of annotated reference genes. However, functional annotation is markedly less sensitive to the specific set of reference genomes available than the methods discussed above, since any gene with detectable homology will suffice. Moreover, one can easily imagine a different implementation that clusters the reads contained in the samples themselves without identifying specific orthology groups, making this approach entirely independent from any exogenous genomic data (see also below). These properties of metagenomic deconvolution make it an ideal framework for analyzing metagenomic samples from the many microbial habitats yet to be extensively characterized.

A deconvolution-based framework also has some obvious limitations. First, it requires multiple metagenomic samples and information on both taxonomic and gene abundances. While this may have been a significantly limiting factor in the past, with the ever decreasing cost of sequencing technologies and the recently introduced advances in molecular and computational profiling of taxonomic and gene compositions, current studies in metagenomics often generate such data regardless of planned downstream analyses (e.g., [6], [7]). Furthermore, if a genomic element is known to be sparsely distributed among the taxa in a collection of samples, then regularized regression techniques, such as the lasso [56], can be used to predict the presence and absence of the genomic element among the taxa, even if the number of samples is much smaller than the number of taxa. Additionally, as demonstrated above, strong correlations between taxa abundances reduce the amount of variation, decreasing the signal and potentially hindering the accuracy of the deconvolution process. Improved understanding of the assembly rules that give rise to such correlations may help alleviate this problem. Finally, our framework relies on accurate estimations of gene and taxonomic abundances. These estimations may be skewed by annotation errors or by the specific method used to evaluate relative taxonomic abundances. Specifically, 16S copy number variation between taxa in a sample (even between strains of the same species [65]) may markedly bias abundance estimates, although this can largely be resolved by estimating the 16S copy number in each taxon using measured copy numbers in sequenced strains [66]. No such correction was performed in this study, as we sought to present a generic implementation of the metagenomic deconvolution framework applicable to analyzing sets of metagenomic samples without the need for coverage by reference genomes.

The deconvolution framework presented in this study can serve as a basis for many exciting extensions and can be integrated with other analysis methods. It is easy, for example, to redefine the scale at which both genomic elements and taxa are defined. In analyzing the HMP samples, we partition genes among genera, rather than into individual OTUs. A similar approach can be used to deconvolve higher or lower (e.g., strain) phylogenetic levels or even to deconvolve different taxa at different phylogenetic levels. One can, for example, target particular species for genome reconstruction while resolving others only on the genus level. Similarly, deconvolution can be performed for other genomic elements such as k-mers or other discrete sequence motifs. Deconvolution can also be carried out incrementally, first deconvolving highly abundant taxa or taxa for which partial genomic information is available. The expected contribution of each deconvolved taxon to the overall gene count in the metagenome can then be calculated and subtracted computationally from each sample, effectively generating lower complexity samples and facilitating the deconvolution of additional taxa. A similar approach can also be used to subtract the contribution of fully sequenced strains whose genomic content is known. Notably, in implementing and characterizing the deconvolution framework here, we did not utilize any information about known strains' genomes. Such information can be used in principal to calibrate various parameters and to normalize the obtained results. Most importantly, this metagenomic deconvolution framework can be naturally combined with other binning methods or metagenomic assembly efforts [67]. For example, by treating contigs, or groups of contigs (such as those generated by metagenomic linkage groups [12] or metagenomic clusters [30]) as individual genomic elements (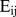 and 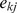, Eq. 3), deconvolution can be used to assign these larger-scale genomic fragments to individual taxa and aid in assembly. Such a process would be especially useful in the case of time-series data where the abundances of strains change with time. Finally, the metagenomic deconvolution framework facilitates novel analysis approaches for studying microbial communities. Samples taken from a community can be post-processed in multiple ways to preferentially select for certain taxa (e.g. filter microbes by size or nutrient requirements), essentially creating different views of the same community. Deconvolution can then be used to recombine these views and to reconstruct the genomic content of each taxon.

To truly take advantage of the data being produced by metagenomic studies and by forthcoming studies of the metatranscriptome and metametabolome of many microbial communities, tools that can reliably determine the taxonomic origins of each “meta'omic” element are crucial. Metagenomic deconvolution represents both a novel strategy for the analysis of such meta'omic data and a framework for future developments in genome reconstruction and annotation.

## Methods

### Simple synthetic metagenomic samples

Simple models of metagenomic samples were created from collections of model “microbial species” by simulating genomes and shotgun sequencing without the complexities of actual genome sequences or sequencing error. Microbial species were modeled as a set of “genes”, taken from a global set of 100 gene orthology groups (simply referred to as genes). These genes had no sequence; their only property was length, which was chosen at random between 400 and 500 bases, and was fixed across all homologs. Simulations with species-specific variations in gene length showed qualitatively similar results (see Supporting Text S1). Each of the 100 genes was randomly assigned to between 20 to 80% of the species, with each species containing a minimum of 10 genes. Within a given species, each gene had a 5% chance of duplication, with the rates for higher copy number decreasing exponentially. Each species included a single copy of a “constant gene” with a length of 1500 bases (see Results).

Sets of model “microbial communities” were created as a linear combination of model microbial species. Each microbial community in a set had a different, but correlated, species abundance profile, with the abundance of a species *j* in sample *i*, determined by the function, 
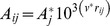
, where 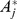 represents the typical abundance of species *j*, *v* is a parameter that governs the amount of inter-sample correlation in the abundance profiles and *r_ij_* is a Gaussian-distributed random number with mean of 0 and standard deviation of 1. To examine the robustness of deconvolution to the number of species and the level of inter-sample correlation, 30 different sets of related communities were created, with the number of species ranging from 20 to 100 in steps of 20, and the correlation parameter *v* logarithmically distributed, 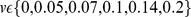 (Figure S4). The set of communities analyzed in the main text was modeled with 60 species and a correlation parameter of *v* = 0.10.

Model metagenomic samples were generated from each microbial community by simulating a shotgun sequencing sampling: Sequencing reads were created by randomly selecting a gene in the community, weighted by the relative abundance of each gene in the community (Eq. 3). 5M sequencing reads were generated for each community. Due to the finite sequencing depth and the exponentially distributed species abundances, species whose abundances were below 0.5% of the most abundant species in the sample were considered absent from the set of shotgun metagenomic reads and excluded from our analysis. These samples and the related data can be found in Supporting Dataset S1 and on our website (http://elbo.gs.washington.edu/download.html).

Deconvolution was performed for species that were present in at least half the samples using least squares, non-negative least squares, and lasso regression using the solvers implemented in MATLAB. The computation times for these deconvolution runs on a four-core 3.10 GHz Intel Xeon CPU were 2±1×10^−4^ s/gene, 4.6±0.8×10^−3^ s/gene, and 1.63±0.05 s/gene for least squares, non-negative least squares, and lasso regression respectively. Adding additional samples required 8×10^−7^, 7×10^−7^, and 0.9 s/gene/sample for least squares, non-negative least squares, and lasso (for underdetermined systems) regression, respectively; for overdetermined systems, lasso had a performance increase of 1.7×10^−2^ s/gene/sample. Adding additional species required 2×10^−6^, 7×10^−5^, and 4×10^−2^ s/gene/species for least squares, non-negative least squares, and lasso regression, respectively.

### Synthetic metagenomic samples with sequencing and annotation error

Simple models of metagenomic samples were created from the fully sequenced genomes of microbial reference organisms to introduce the complexities associated with actual genome sequences and annotation error. 10 model communities were composed as linear combinations of the reference organisms *Alistipes shahii* WAL 8301, *Ruminococcus champanellensis* sp. nov., and *Bifidobacterium longum longum* F8. These strains were chosen because they each had a different level of coverage by the KEGG database used in this study (see below): *B. Longum* had a different strain of the same species present in the database; *R. Champanellensis* had only a member of the same genus present; and *A. Shahii* had no relatives within the same genus present. Complete species genomes were obtained from the Integrated Microbial Genomes database [68]. These communities had species relative abundances assigned randomly, ranging over a thousand-fold; however, the magnitude of the range of relative abundances was shown to have little impact on our results (Supporting Text S1). Model metagenomic samples were created from each community by simulating 1M shotgun metagenomic sequencing reads with Metasim [50], using 80-base reads with an Illumina sequencing error model.

The abundances of gene orthology groups present in each model metagenomic sample were determined from the set of reads by annotating each read with KEGG orthology groups (KOs) through a translated BLAST search against the KEGG Orthology v60 [19]. Reads were annotated with the KO of the best hit with an E-value<1, similar to the method employed by the HMP [6]. Reads with a best-hit match to a KEGG gene without a KO annotation were not assigned a KO. In cases of e-value ties, the read was assigned the annotations of all the tied matches, with each annotation receiving a fractional count. Reads containing an ambiguous base were not annotated. The abundance of the 16S rRNA KO was determined through a nucleotide BLAST search against a custom database containing the sequences of all 16S rRNA genes in the KEGG database. These samples (as well as the 20 strain community samples) and the related data can be found in Supporting Dataset S2, Supporting Dataset S3, and on our website (http://elbo.gs.washington.edu/download.html).

Deconvolution was performed using least squares, non-negative least squares, and lasso regression for KOs whose average count was greater than 0.1% of the most abundant KO using the solvers implemented in MATLAB. The computation times for these deconvolution runs on a four-core 3.10 GHz Intel Xeon CPU were 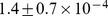 s/KO, 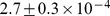 s/KO, and 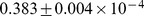 s/KO for least squares, non-negative least squares, and lasso regression respectively.

To evaluate the presence/absence prediction made by our framework, we used a null model in which community members are all assumed to have an identical (‘convoluted’) genome, directly derived from the set of metagenomic samples. Specifically, the KO lengths in this model corresponded to the average relative abundance of each KO across all samples, normalized by the length and abundance of the 16S KO. Formally, the length of KO *j*, 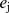, was calculated as 
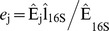
, where 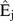 is the average relative abundance of KO *j* across all metagenomic samples, 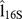 is the average length of the 16S KO, and 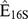 is the average relative abundance of the 16S KO.

### Human Microbiome Project datasets

HMP data was downloaded from the HMP Data Analysis and Coordination Center (DACC) (http://www.hmpdacc.org/). OTU abundances and taxonomy were based on the QIIME 16S pipeline [14]. The abundance of each genus was calculated by adding the abundances of all OTUs in that genus. Only genera with relative abundance >5% in at least one sample were considered. KO abundances were based on the HUMAnN pipeline [19]. For samples with technical replicates, the replicate with the greater sequencing depth was used. To reduce annotation error, only KOs present in at least 80% of the tongue dorsum samples were used in the analysis. Since HMP KO abundance data included only proteins, we used a set of 15 ribosomal proteins ubiquitous across Bacteria and Archaea instead of the 16S RNA gene as the constant genomic element in Eq. 5 (see below). Deconvolution was performed for KOs that were present in at least half the samples using least squares, non-negative least squares, and lasso regression using the solvers implemented in MATLAB. The computation times for these deconvolution runs on a four-core 3.10 GHz Intel Xeon CPU were 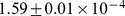 s/KO, 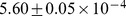 s/KO, and 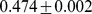 s/KO for least squares, non-negative least squares, and lasso regression respectively.

### Human Microbiome Project reference genomes

Genomes for the HMP Reference Organisms were obtained from the Integrated Microbial Genomes – Human Microbiome Project (IMG/HMP) database on 5/7/2012 (http://www.hmpdacc-resources.org/cgi-bin/imgm_hmp/main.cgi). In order for the annotations to be compatible with the version of the database used in this study, each organism was annotated through a BLAST search of each ORF against the KEGG genes database with a protocol similar to that used by the IMG [68]. Each ORF was annotated with the KO of the best match gene with an e-value <1×10^−5^. In cases of ties, the ORF was annotated with all corresponding KOs, with a proportionally fractional count. ORFs that best matched a KEGG gene with no KO annotation were not annotated. KOs were considered to be present in a genome if this annotation procedure resulted in a copy number ≥0.1. For species with more than one sequenced strain, the average annotation across strains was used. KOs present in at least 75% of HMP reference organisms were considered core KOs and were removed from the analysis. Similarly, KOs present in fewer than 1% of HMP reference genomes were assumed to be spurious annotations and were excluded.

### Selection of ribosomal genes as constant genomic elements

One of the components required to deconvolve metagenomic samples is a constant genomic element or gene that can be used as a normalization coefficient for inferring the length (or copy number) of all other genomic elements. Ideally, genes used for normalization should be present in all the species in the community, have the same copy number in each genome, and have a consistent length across all species. The 16S rRNA gene is a natural candidate, but other gene orthology groups can be used as well. Specifically, in the main text, we deconvolved tongue dorsum samples from the Human Microbiome Project using a combination of ribosomal protein-coding genes. Ribosomal genes are generally good candidates for normalization since the ribosome is a highly-conserved construct. Using the combined abundances of multiple genes can reduce the potentially deleterious effect of read annotation errors in any one gene. Starting with 31 ribosomal protein-coding KOs present in both bacteria and archaea, we first considered those that were present in at least 1445 (98%) of the 1475 bacteria and archaea in KEGG v60 [19]. Of these KOs, we selected a subset of 15 KOs that had a lower variation in length across all genomes than the 16S gene (Table S1). These 15 KOs were used jointly as our constant genomic element for normalization, using the sum of the abundances as the constant genomic element abundance 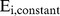 and sum of the lengths as the constant genomic element length 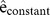 in Eq. 5.

## Supporting Information

Dataset S1 Species gene lengths and species and gene abundances for each sample and for each simulated dataset modeled without sequencing and annotation error. The number of species and parameter ν are given for each dataset. Gene 100 corresponds to the constant copy number gene used to normalize samples.
(XLS)

Dataset S2 Strain KO lengths, strain and KO abundances for each sample, and the observed annotation error for the dataset simulated with sequencing and annotation error.
(XLS)

Dataset S3 Strain KO lengths, and strain and KO abundances for each sample for the simulated dataset based on HMP Mock Community B. Note that strain abundances are given as the apparent abundances generated from the relative abundances of 16S genes, equivalent to the actual abundances multiplied by the copy numbers.
(XLS)

Figure S1 Schematic of methods for grouping sequencing reads or genomic elements found in shotgun metagenomic sequencing data. Sequencing reads are shown in gray. (**A**) Alignment-based methods map reads to a set of reference genomes (red and blue). (**B**) Taxonomic classification methods assign higher-level phylogenetic labels (light red and blue) to each read through sequence homology searches. (**C**) Assembly-based methods physically link reads into contigs and scaffolds (light red and blue) using sequence overlap and paired-end information. (**D**) Binning methods exclusively cluster reads or genomic elements into a discrete number of groups (blue and red dashed circles). (**E**) Deconvolution-based approaches create groupings (red and blue) of genomic elements (green and orange) that best explain the observed samples.
(TIF)

Figure S2 The abundance profiles of 60 species in 100 samples generated by a simple model of microbial communities. Each color represents the abundance of one species.
(TIF)

Figure S3 Accuracy and recall for predicting the presence of genes in species from synthetic metagenomic samples as a function of the threshold used. Threshold values are represented as the ratio between the predicted length and the average length across sequenced genomes.
(TIF)

Figure S4 Species abundance profiles for 30 sets of synthetic communities, with varying levels of inter-sample correlation (x-axis) and varying number of species (y-axis). Each color represents the abundance of one species. The inter-sample correlation (parameterized as ν) represents the level at which the species abundance profile varies between samples, with ν = 0 corresponding to zero variation and perfect correlation, and the level of variation increasing exponentially with ν (see Methods).
(TIF)

Figure S5 The impact of the number of species in the community and of correlations between species abundances on metagenomic deconvolution. (**A**) Accuracy and (**B**) recall in predicting the presence of genes as a function of the level of inter-sample correlation (see Figure S4 and Methods) and for different numbers of species in the community. Note that the effect of the number of species is dwarfed by the effect of abundance correlations between species. As in the main text, a threshold of 0.5 of the gene length was used.
(TIF)

Figure S6 KO read relative abundance as obtained by a translated BLAST search vs. actual KO relative abundances averaged across all samples. The 16S genes are highlighted for comparison.
(TIF)

Figure S7 Predicted KO lengths vs. actual KO lengths, assuming perfect annotation. Compare to Figure 3.
(TIF)

Figure S8 Reconstructing the genomic content of reference genomes from simulated mixed metagenomic samples based on the HMP Mock Community using metagenomic deconvolution. ROC curves (solid line; AUC = 0.87) for predicting KO presence and absence across all species as a function of the threshold used to predict the presence of a KO. ROC curve for a naïve convolved prediction (dashed line; AUC = 0.77) is illustrated for comparison.
(TIF)

Figure S9 Comparison of alternative regression methods for deconvolving the HMP tongue dorsum samples. The average similarity in KO content between each reconstructed genus and sequenced genomes from the various genera using least squares regression (**A**) and lasso (**B**). Similarity metric and parameters are as in Figure 5.
(TIF)

Figure S10 Performance of a simple correlation-based heuristic for predicting the genomic content of species from synthetic metagenomic samples. (**A**) Accuracy and recall for predicting the presence of genes in species from synthetic metagenomic samples using the naïve correlation-based method as a function of the correlation coefficient threshold used. (**B**) Accuracy and recall for predicting the presence of genes in species from synthetic metagenomic samples using the naïve correlation-based method as a function of the level of inter-sample correlation (see Figure S4 and Methods). Results using the Pearson correlation are shown (using a Spearman correlation had little effect on the results; Supporting Text S1).
(TIF)

Figure S11 Comparison of alternative regression methods for metagenomic deconvolution. Accuracy of least squares, non-negative least squares, and lasso regression are illustrated for the simple synthetic model (**A**) and for the synthetic model with sequencing and annotation errors (**B**) as a function of the threshold used. Threshold values are represented as the ratio between the predicted length and the average length across sequenced genomes.
(TIF)

Figure S12 Accuracy and recall for predicting the presence of genes in species from synthetic metagenomic samples where species were given unique lengths for each gene as a function of the threshold used. Threshold values are represented as the ratio between the predicted length and the average length across sequenced genomes. Compare to Figure S3.
(TIF)

Table S1 Ribosomal genes used as constant genomic elements in the deconvolution of the Human Microbiome Project datasets.
(XLS)

Table S2 The optimal number of clusters detected for each technique.
(XLS)

Text S1 Supporting information, including an analysis of a simple correlation-based heuristic for predicting the genomic content of microbiome taxa, a comparison of the metagenomic deconvolution framework to existing binning and deconvolution methods, the use of alternative regression methods in metagenomic deconvolution, a reanalysis of the deconvolution of synthetic microbial communities with sequencing and annotation errors for more uniform abundance profiles, the prediction of variable and taxa-specific genes in the synthetic microbial communities with sequencing and annotation errors, the deconvolution of a 20-strain model microbial community based on the HMP mock communities, and a reanalysis of the deconvolution of the simple synthetic metagenomic samples for the case of species-specific gene lengths.
(DOC)
