# Supplementary figures and images for: Reconstructing the Genomic Content of Microbiome Taxa through Shotgun Metagenomic Deconvolution
Source: PLoS Comput Biol. 2013 Oct 17;9(10):e1003292. doi: 10.1371/journal.pcbi.1003292 (PMC3798274; doi:10.1371/journal.pcbi.1003292)

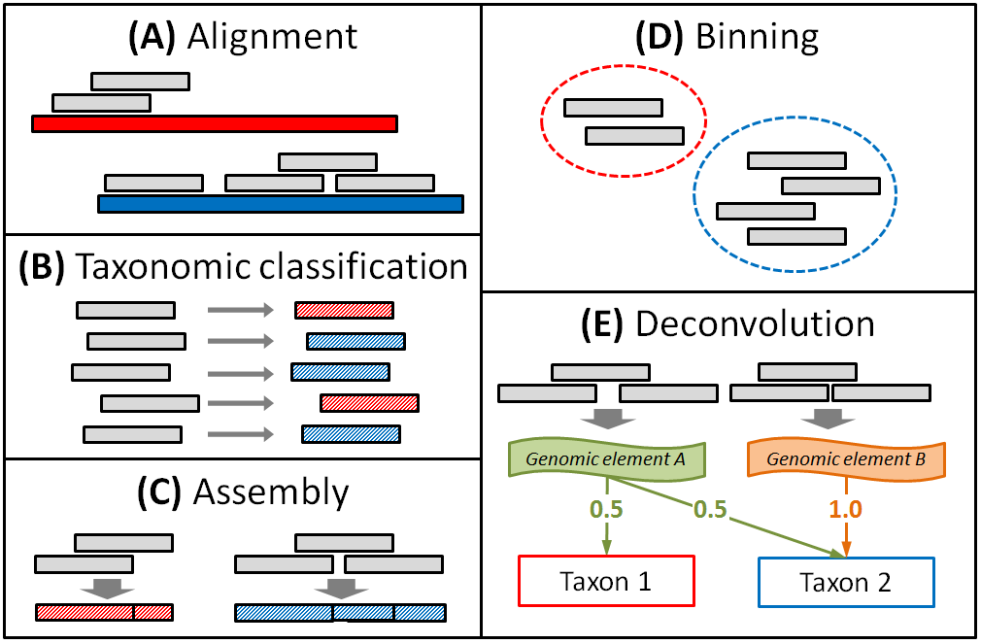

Supplement: Figure S1 — Schematic of methods for grouping sequencing reads or genomic elements found in shotgun metagenomic sequencing data. Sequencing reads are shown in gray. (A) Alignment-based methods map reads to a set of reference genomes (red and blue). (B) Taxonomic classification methods assign higher-level phylogenetic labels (light red and blue) to each read through sequence homology searches. (C) Assembly-based methods physically link reads into contigs and scaffolds (light red and blue) using sequence overlap and paired-end information. (D) Binning methods exclusively cluster reads or genomic elements into a discrete number of groups (blue and red dashed circles). (E) Deconvolution-based approaches create groupings (red and blue) of genomic elements (green and orange) that best explain the observed samples. (TIF) [file pcbi.1003292.s004.tif]

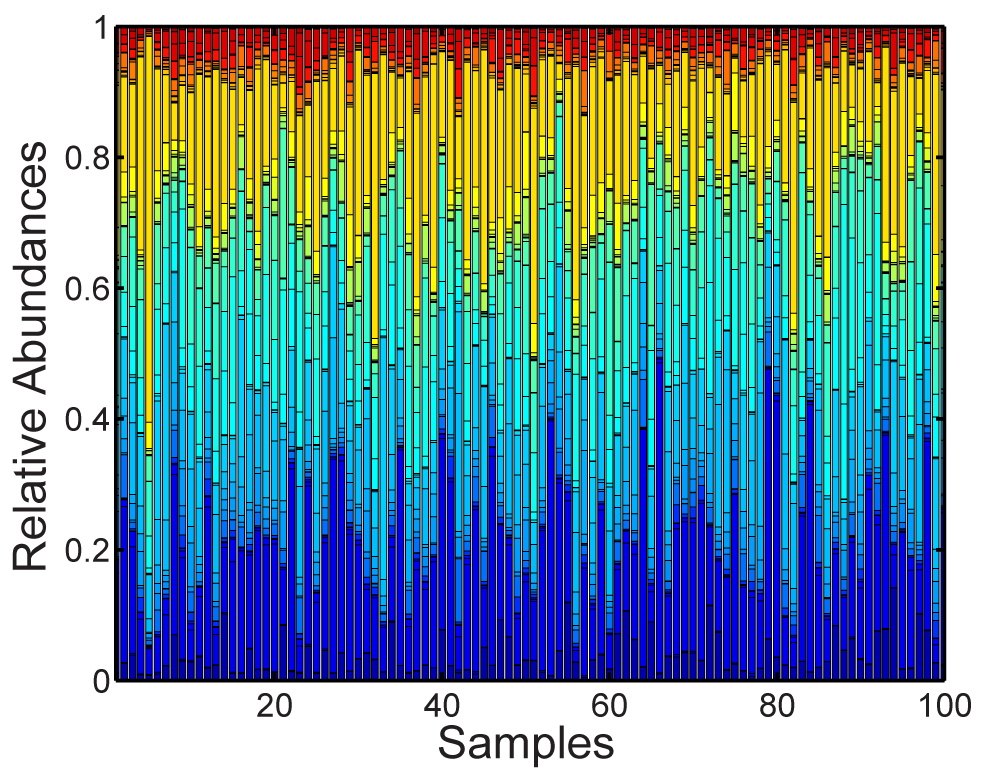

Supplement: Figure S2 — The abundance profiles of 60 species in 100 samples generated by a simple model of microbial communities. Each color represents the abundance of one species. (TIF) [file pcbi.1003292.s005.tif]

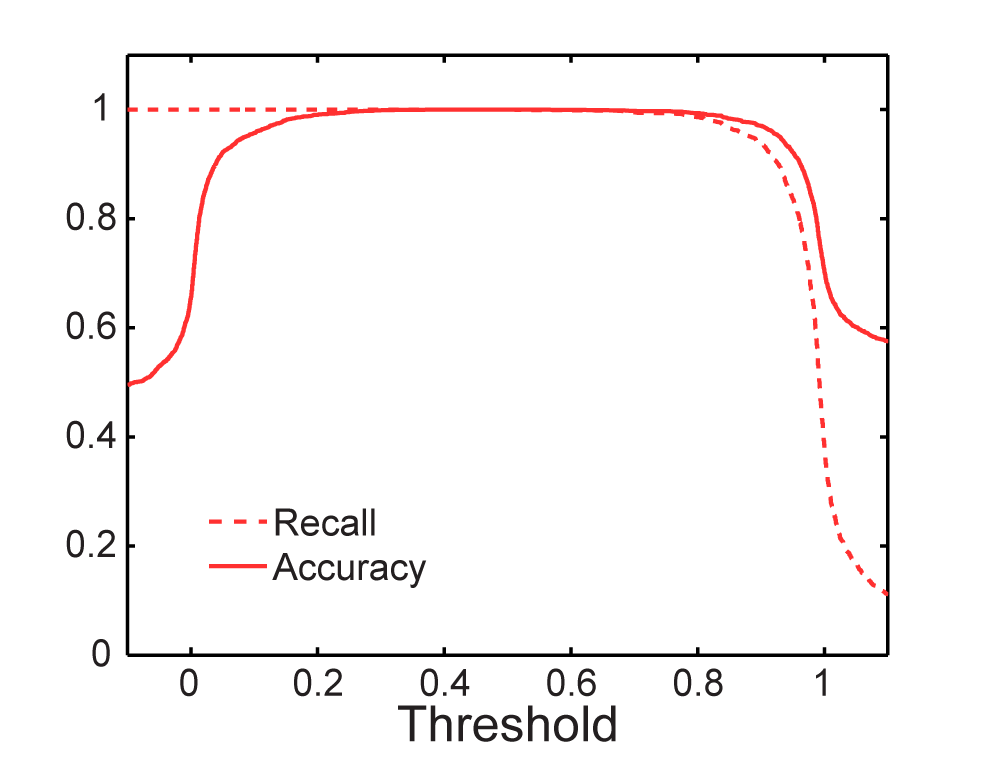

Supplement: Figure S3 — Accuracy and recall for predicting the presence of genes in species from synthetic metagenomic samples as a function of the threshold used. Threshold values are represented as the ratio between the predicted length and the average length across sequenced genomes. (TIF) [file pcbi.1003292.s006.tif]

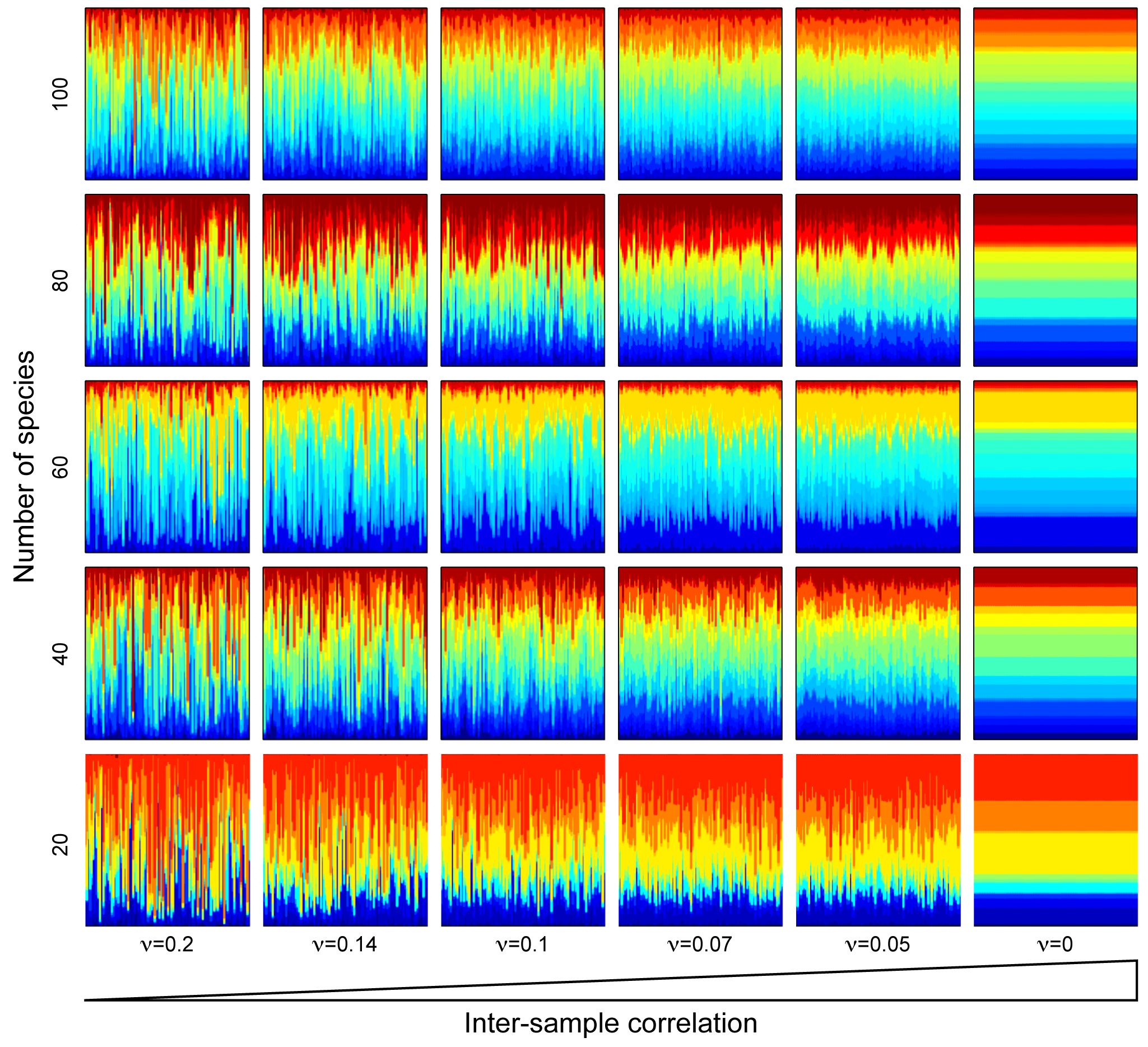

Supplement: Figure S4 — Species abundance profiles for 30 sets of synthetic communities, with varying levels of inter-sample correlation (x-axis) and varying number of species (y-axis). Each color represents the abundance of one species. The inter-sample correlation (parameterized as ν) represents the level at which the species abundance profile varies between samples, with ν = 0 corresponding to zero variation and perfect correlation, and the level of variation increasing exponentially with ν (see Methods). (TIF) [file pcbi.1003292.s007.tif]

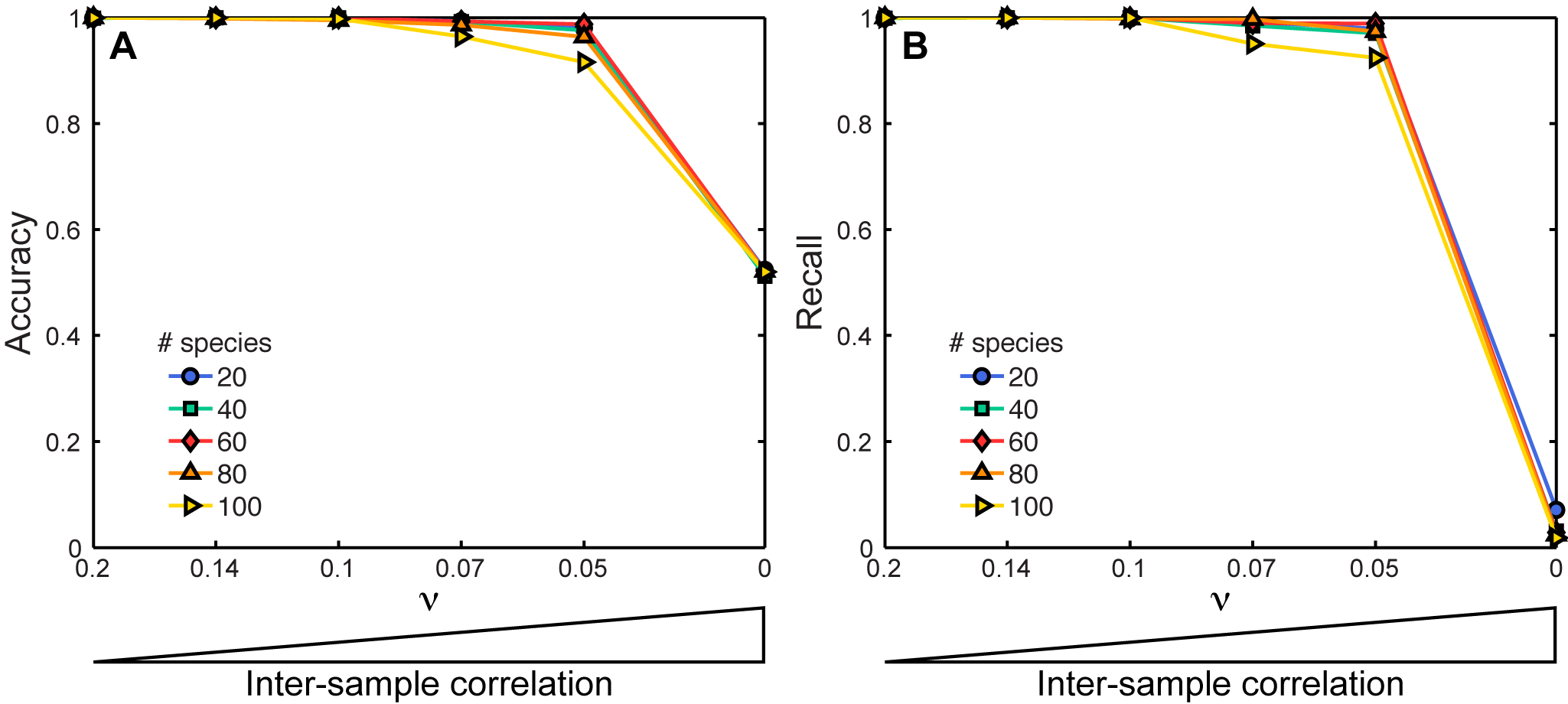

Supplement: Figure S5 — The impact of the number of species in the community and of correlations between species abundances on metagenomic deconvolution. (A) Accuracy and (B) recall in predicting the presence of genes as a function of the level of inter-sample correlation (see Figure S4 and Methods) and for different numbers of species in the community. Note that the effect of the number of species is dwarfed by the effect of abundance correlations between species. As in the main text, a threshold of 0.5 of the gene length was used. (TIF) [file pcbi.1003292.s008.tif]

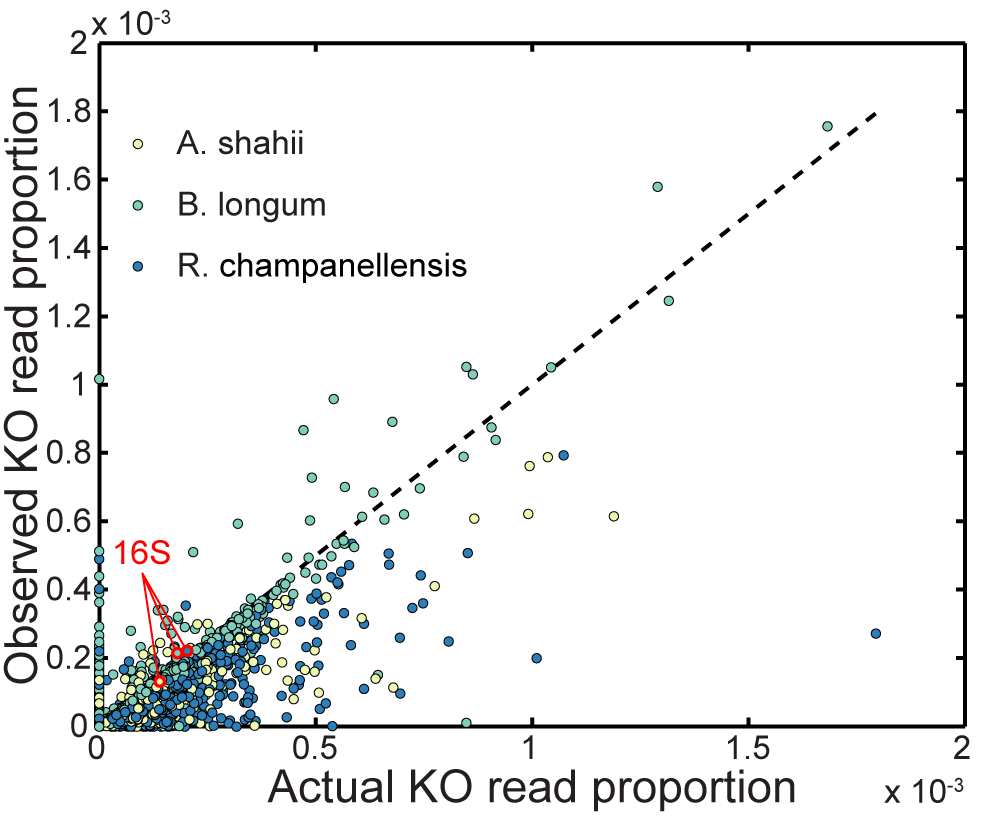

Supplement: Figure S6 — KO read relative abundance as obtained by a translated BLAST search vs. actual KO relative abundances averaged across all samples. The 16S genes are highlighted for comparison. (TIF) [file pcbi.1003292.s009.tif]

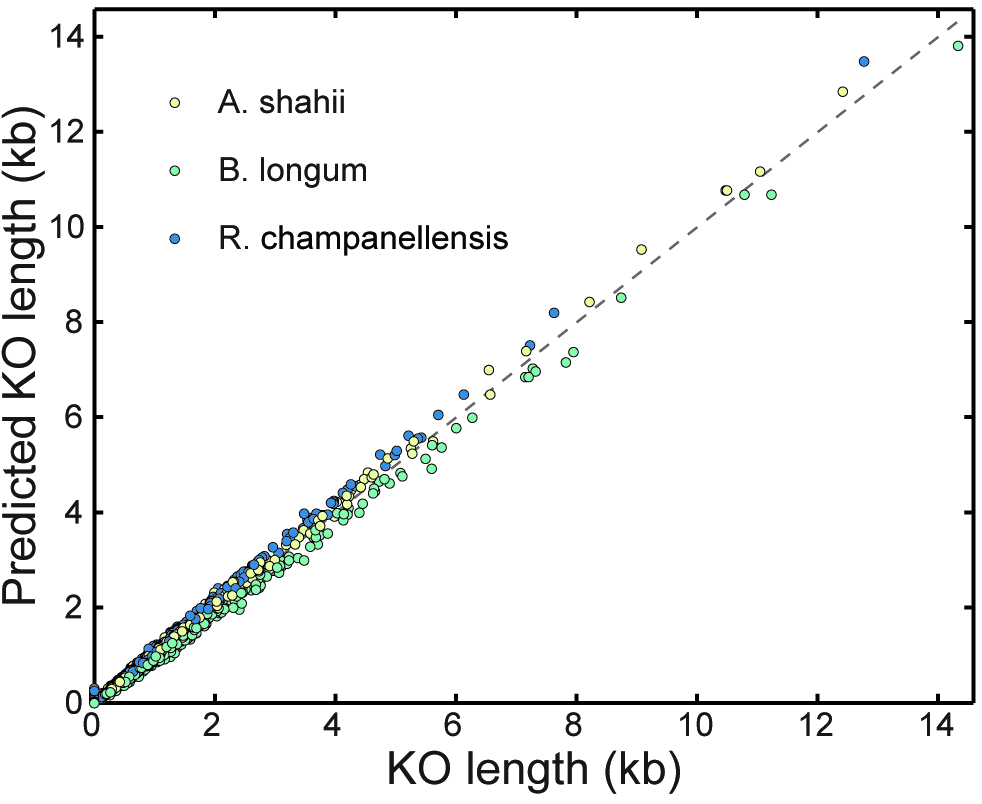

Supplement: Figure S7 — Predicted KO lengths vs. actual KO lengths, assuming perfect annotation. Compare to Figure 3. (TIF) [file pcbi.1003292.s010.tif]

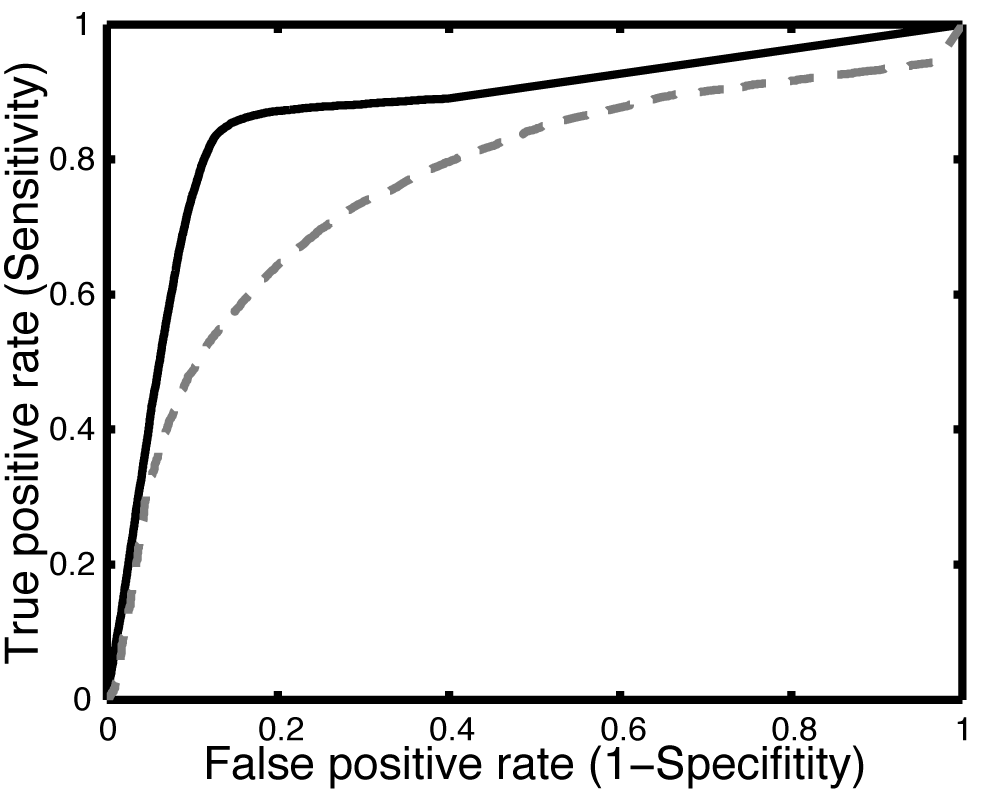

Supplement: Figure S8 — Reconstructing the genomic content of reference genomes from simulated mixed metagenomic samples based on the HMP Mock Community using metagenomic deconvolution. ROC curves (solid line; AUC = 0.87) for predicting KO presence and absence across all species as a function of the threshold used to predict the presence of a KO. ROC curve for a naïve convolved prediction (dashed line; AUC = 0.77) is illustrated for comparison. (TIF) [file pcbi.1003292.s011.tif]

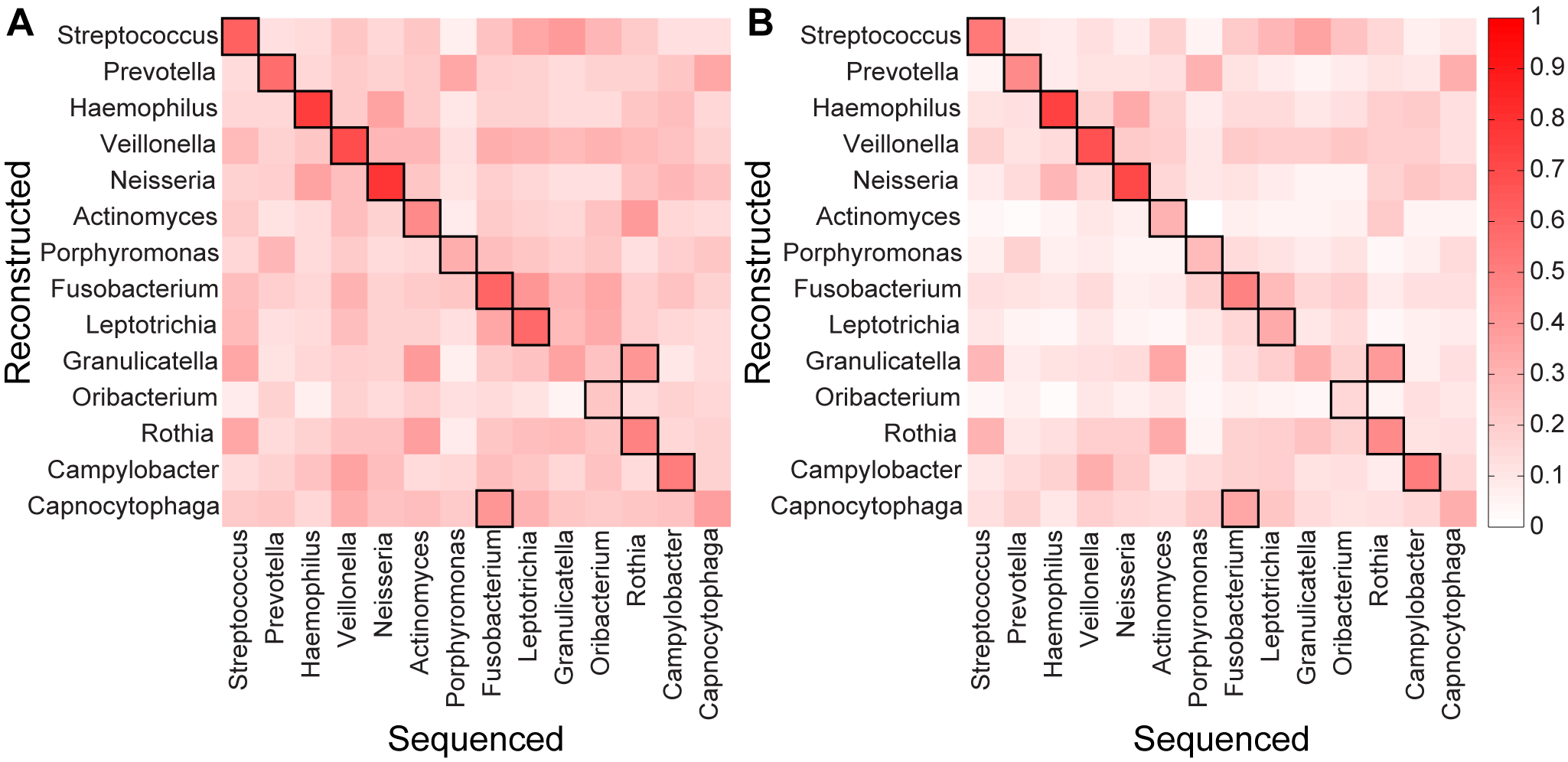

Supplement: Figure S9 — Comparison of alternative regression methods for deconvolving the HMP tongue dorsum samples. The average similarity in KO content between each reconstructed genus and sequenced genomes from the various genera using least squares regression (A) and lasso (B). Similarity metric and parameters are as in Figure 5. (TIF) [file pcbi.1003292.s012.tif]

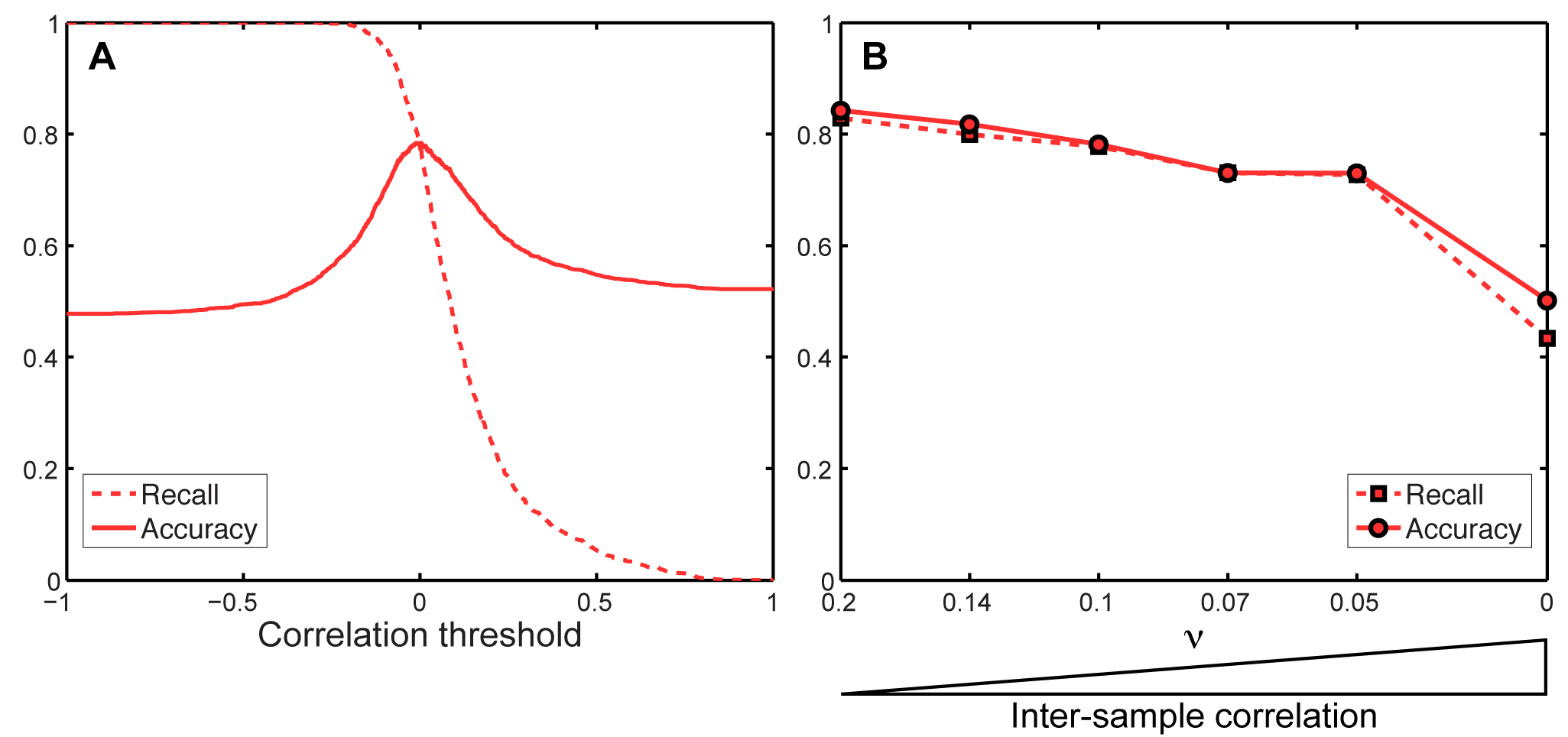

Supplement: Figure S10 — Performance of a simple correlation-based heuristic for predicting the genomic content of species from synthetic metagenomic samples. (A) Accuracy and recall for predicting the presence of genes in species from synthetic metagenomic samples using the naïve correlation-based method as a function of the correlation coefficient threshold used. (B) Accuracy and recall for predicting the presence of genes in species from synthetic metagenomic samples using the naïve correlation-based method as a function of the level of inter-sample correlation (see Figure S4 and Methods). Results using the Pearson correlation are shown (using a Spearman correlation had little effect on the results; Supporting Text S1). (TIF) [file pcbi.1003292.s013.tif]

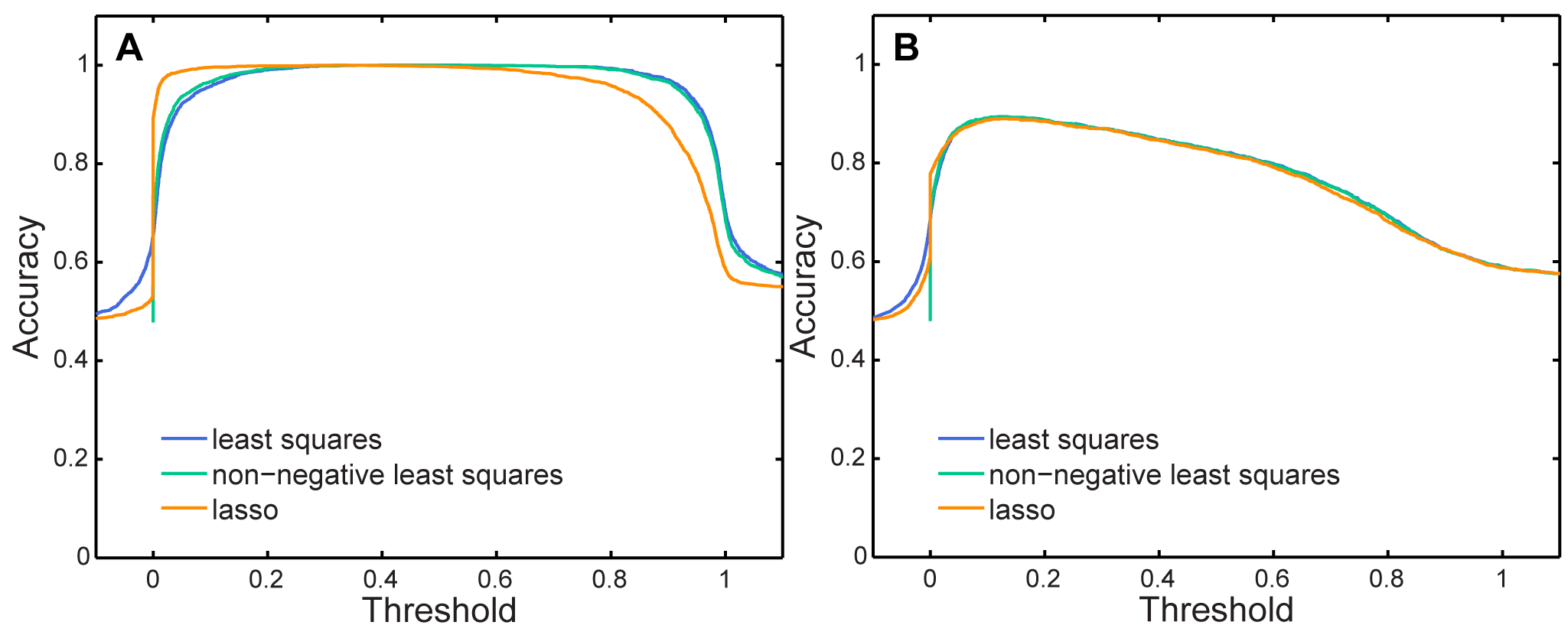

Supplement: Figure S11 — Comparison of alternative regression methods for metagenomic deconvolution. Accuracy of least squares, non-negative least squares, and lasso regression are illustrated for the simple synthetic model (A) and for the synthetic model with sequencing and annotation errors (B) as a function of the threshold used. Threshold values are represented as the ratio between the predicted length and the average length across sequenced genomes. (TIF) [file pcbi.1003292.s014.tif]

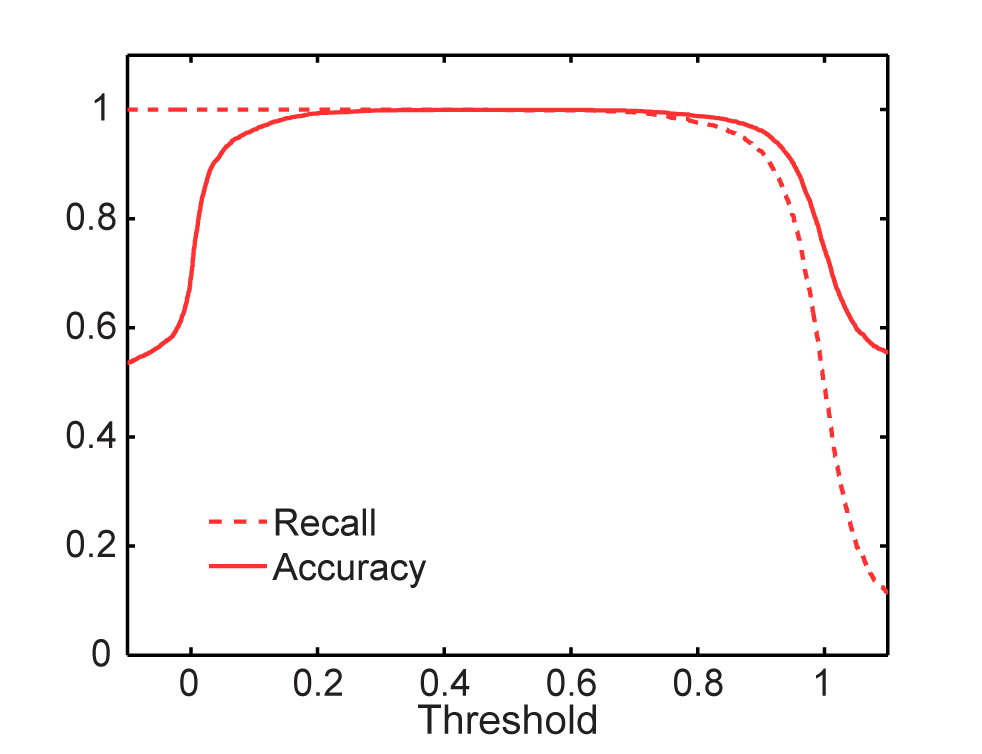

Supplement: Figure S12 — Accuracy and recall for predicting the presence of genes in species from synthetic metagenomic samples where species were given unique lengths for each gene as a function of the threshold used. Threshold values are represented as the ratio between the predicted length and the average length across sequenced genomes. Compare to Figure S3. (TIF) [file pcbi.1003292.s015.tif]
