# Supplementary material for: Reconstructing the Genomic Content of Microbiome Taxa through Shotgun Metagenomic Deconvolution
Source: PLoS Comput Biol. 2013 Oct 17;9(10):e1003292. doi: 10.1371/journal.pcbi.1003292 (PMC3798274; doi:10.1371/journal.pcbi.1003292)
Supplement: Text S1 — Supporting information, including an analysis of a simple correlation-based heuristic for predicting the genomic content of microbiome taxa, a comparison of the metagenomic deconvolution framework to existing binning and deconvolution methods, the use of alternative regression methods in metagenomic deconvolution, a reanalysis of the deconvolution of synthetic microbial communities with sequencing and annotation errors for more uniform abundance profiles, the prediction of variable and taxa-specific genes in the synthetic microbial communities with sequencing and annotation errors, the deconvolution of a 20-strain model microbial community based on the HMP mock communities, and a reanalysis of the deconvolution of the simple synthetic metagenomic samples for the case of species-specific gene lengths. (DOC) [file pcbi.1003292.s018.doc]

Supporting Text: Reconstructing the Genomic Content of Microbiome Taxa through Shotgun Metagenomic Deconvolution

Rogan Carr^1^, Shai S. Shen-Orr^2^, and Elhanan Borenstein^1,3,4*^

^1^ Department of Genome Sciences, University of Washington, Seattle, Washington, U.S.A.

^2^ Department of Immunology, Rappaport Institute of Medical Research, Faculty of Medicine and Faculty of Biology, Technion, Haifa, Israel.

^3^ Department of Computer Science and Engineering, University of Washington, Seattle, Washington, U.S.A.

^4^ Santa Fe Institute, Santa Fe, New Mexico, U.S.A.

* Corresponding author, elbo@uw.edu

# Naïve correlation-based reconstruction of genomic content

At its heart, metagenomic deconvolution compares variation in the abundances of genomic elements to variation in the abundances of species across metagenomic samples to determine the associations between genomic elements and species. A naïve method to determine such associations can therefore rely on a simple calculation of the correlation between the abundances of each genomic element and those of each species. Genomic elements whose abundances are highly correlated across the samples with the abundances of a given species will then be assumed to be present in that species’ genome. This method ignores potential contributions to the abundances of these elements from other species and can therefore provide a baseline for evaluating the success of more involved methods such as metagenomic deconvolution.

To evaluate this naïve method, we applied it to the synthetic metagenomic samples analyzed in the main text. For each species, we calculated the Pearson and Spearman correlation coefficients between its abundances across the metagenomic samples and the abundances of each genomic element. To predict the presence or absence of a genomic element in a species, we used a simple threshold-based approach, considering any element for which the correlation coefficient exceeded a predefined threshold to be present in the species’ genome. Applying the threshold for which the correlation method performed best (a threshold of 0) and using the Pearson correlation, we found that this simple correlation-based method had an accuracy of 78% and recall of 78% (Figure S10). Using the Spearman correlation had little impact on performances, with an accuracy of 77% and recall of 77%. However, as demonstrated in Figure S10A, this approach was extremely sensitive to slight variations in the threshold value used compared to deconvolution-based methods (see Figures S3 and S11A for comparison). Moreover, one obvious limitation of this method is that it treats each species independently. Indeed, when this correlation-based method was applied to communities with higher inter-sample correlations (also giving rise to higher inter-species correlations), we found that the accuracy and recall of reconstructed genomes markedly decreased (Figure S10B; compare with Figure S5B for deconvolution).

# Comparison to existing binning and deconvolution techniques

In this section, we have compared the metagenomic deconvolution framework to existing multi-sample binning and deconvolution strategies to highlight the core differences between the approaches. We used the simulated metagenomic samples incorporating sequencing and annotation error, in which the genomic elements are KOs (genes) that originate from one, two or all three strains (Figure 4B; see Results and Methods for a complete description of these datasets). Genomic reconstructions created with the metagenomic deconvolution framework were compared to the predictions given by k-means clustering (representing a generic binning scheme), k-medoids clustering (representing MetaBin [1]), CHAMELEON clustering [2] (representing the metagenomic linkage group analysis, MLG [3]), the markov clustering algorithm (MCL [4]; representing metagenomic clustering analysis, MGC [5]), and non-negative matrix factorization (NMF [6–8]). The metagenomic binning techniques MetaBin and MLG have protocols particular to the types of data they were developed to analyze. For this comparison we therefore implemented their core technologies as a proxy for the performances of these methods.

The dataset here was represented by a matrix whose entries are the relative abundance of each KO in each sample. Using this matrix as an input, the binning and deconvolution analyses were performed as follows: K-means clustering was implemented using the “kmeans” function in the MATLAB Statistics Toolbox. K-medoids clustering was implemented using the “K-medoids” MATLAB package [9]. The CHAMELEON algorithm was implemented using a custom MATLAB code and the METIS software package [10]. CHAMELEON clustering was performed with a k-nearest-neighbor distance of 10, and the number of initial sub-clusters taken as the square root of the number of KOs in the sample, rounded to the nearest integer. For the second phase of the CHAMELEON process, an alpha of 1 and a similarity index of 0.4 were used, in accordance with the MLG protocol [3].To recreate the MCG protocol, we constructed networks by placing an edge between KOs with a Pearson correlation (rho) of at least 0.85 using a distance of 1-rho, and three different MCL calculations were performed using inflation values of 1.4, 2.0, and 6.0, in accordance with the MCG manuscript [5]. MCL was implemented using custom MATLAB code and MCL-Edge (http://micans.org/mcl/). NMF was implemented using the NMF MATLAB Toolbox [11]. To calculate the approximation matrices W and H, we chose the factorization that minimized the KL-divergence between the KO abundance matrix X and the product W×H, the protocol used in refs [7,8]. For each prediction, 10 or 100 replicate calculations were performed for CHAMELEON and NMF, or k-means and k-medoids, respectively. For the clustering algorithms, the solution with the least within sum-of-squares (WSS) distance (the sum of all squared distances between each point and its cluster’s mean (or medoid)) was chosen, while the solution with the lowest residual was chosen for NMF.

All the methods being compared, including metagenomic deconvolution, partition the KOs into groups, here representing the individual strains making up the metagenomic samples, based on the similarity of their abundances across the samples. As discussed in the main text (see Figure S1 for a visual representation), the clustering algorithms presented here, including MLG and MCL, are “binning” techniques that exclusively cluster each KO into a single group, while NMF is a deconvolution procedure that allows KOs to belong to multiple groups. The CHAMELEON algorithm and MCL automatically detect the number of such groupings in the set of samples, while k-means clustering, k-medoids clustering and NMF take the number of groupings as an input, usually determined by comparing a summary statistic as a function of the number of clusters. To determine the optimal number of clusters for the k-means and k-medoids clustering algorithms, we calculated the within-sum-of-square (WSS) distances as a function of the number of clusters and chose the number of clusters for which adding another cluster negligibly reduced the WSS. To calculate the optimal number of groupings for NMF, we calculated the “concordance index” as a function of the number of features, and chose the solution with the highest concordance [7,8]. None of the methods we tested detected the same number of groupings as there were strains in the samples, even when only the KOs that belonged to a single strain were used in the analysis. The number of clusters detected by each method varied and was method and parameter-specific (Table S2). The optimal solutions for these methods could thus not be directly used to reconstruct the genome content for the three strains.

Rather than detecting the number of groups in the metagenomic samples, as is done in practice, the number of groups present in the metagenomic samples can be assumed to be known, for example from 16S sequencing. If we force the number of groups to be equal to the number of strains, solutions representing genomic reconstructions of the three strains can be constructed using k-means, k-medoids, and NMF. (Note that this operation cannot be performed with the CHAMELEON and MCL algorithms underlying MLG and MGC, respectively, because they are data discovery techniques and do not take the number of desired groups as an input.) The genomic reconstructions created in this manner are simply groupings of KOs and are not labeled with a strain. By assigning genomic reconstructions to strains in such a way as to maximize the total reconstruction accuracy, we find that the k-means and k-medoids clustering algorithms had accuracies of 53%, while NMF had an accuracy 89%, comparable to the metagenomic deconvolution. Note that these accuracies represent an upper bound, as the process used to assign species labels to groups is unrealistic for actual metagenomic samples. Moreover, it is not surprising that NMF had comparable accuracy to the metagenomic deconvolution framework, as these two methods perform a mathematically similar operation. However, these methods differ fundamentally in that the quantities calculated by metagenomic deconvolution have a direct physical interpretation, while the NMF quantities represent associations with no direct physical interpretation, and the metagenomic deconvolution framework can solve for each genomic element independently, while NMF solves for the entire set of elements simultaneously.

# Alternative regression methods for metagenomic deconvolution

The deconvolution framework models the genomic content of microbial communities as a linear combination of microbial genomes, and applies linear regression to deconvolve these mixed samples into individual genomes. As noted in the main text, any linear regression method can be used in principle to perform this step. Specifically, the analyses presented in the main text employ least squares or non-negative least squares regression (see Methods) to deconvolve samples. Yet, modern regression techniques may offer additional features that can be useful in this context. For example, least squares regression with L1-regularization, or lasso [12], can be beneficial as it implements both continuous shrinkage and variable selection.

To evaluate the performance of the different regression methods in deconvolving metagenomic samples, we applied our deconvolution frameworks to all three datasets discussed in the main text (namely, simple synthetic metagenomic samples, synthetic metagenomic samples with sequencing and annotation errors, and HMP samples) using three different regression methods: least squares, non-negative least squares, and lasso. We found that for the simple synthetic samples, all three techniques had similar accuracy for predicting gene presence and absence and were able to perfectly reconstruct the various genomes. Least squares and non-negative least squares predictions perform similarly as a function of the threshold value used to predict the presence of a gene, while lasso obtained more accurate predictions at lower threshold values (Figure S11A). This finding is not surprising since lasso explicitly attempts to sets gene lengths to zero. For the results presented in the main text we used least squares regression as it is the simplest technique we examined and is therefore ideal for characterizing the performance of our framework without unnecessary complications. Analyzing synthetic metagenomic samples with annotation errors, we again found that the three methods had similar accuracy for predicting gene presence and absence (Figure S11B). Finally, comparing the accuracy obtained with the various regression methods when applied to the HMP tongue dorsum samples represents a challenge since no “correct” genus-level results are available to evaluate the obtained predictions. Yet, we found HHowevaewwtwthat all three methods provide qualitatively similar prediction for the KO content in reconstructed genera (Figure S9). Furthermore, we found that non-negative least squares and lasso regression had similar accuracy and slightly outperformed least squares regression in predicting the presence and absence of genus-specific KOs (least squares, 0.25 threshold: 89.1%; non-negative least squares, 0.25 threshold: 90.3%; lasso, 0.1 threshold: 90.0%). Using a more stringent definition of genus-specific KOs (see main text), we found a similar pattern (least squares, 0.25 threshold: 90.6%; non-negative least squares, 0.25 threshold: 92.1%; lasso, 0.1 threshold: 91.7%). In the main text we therefore presented results based on the less computationally intensive non-negative least squares regression.

# Deconvolving synthetic communities with more uniform species abundances

In the main text, we applied the deconvolution framework to analyze synthetic metagenomic samples simulated from communities in which species relative abundances ranged a thousand-fold. Such a wide range of abundances, while common in many communities (e.g., [13]), can clearly induce a strong co-variation signal between species and genomic elements, thereby facilitating deconvolution efforts. Here, we therefore aimed to determine whether our framework can still correctly deconvolve metagenomes derived from communities in which variations in species abundances is less extreme. To this end, we generated a set of communities identical to those studied in the main text, but with species abundances chosen randomly between 1 and 10. After simulating sequencing and annotation using the same pipeline as that used in the main text, we applied the deconvolution framework to reconstruct the genomic content of each species. We find qualitatively similar results in both KO length and KO content predictions as those obtained for the communities described in the main text; Specifically, using a threshold of 0.1 of the average KO length, genomes were reconstructed with an accuracy of 87% and a recall of 97% (compared to accuracy of 89% and a recall of 98% in the main text). In contrast, the null model we discussed in the main text had an accuracy of 60% and a recall of 95% in reconstructing these genomes.

# Predicting variable and taxa-specific genes in synthetic microbial communities with sequencing and annotation errors

As we noted in the main text, since most microbes share a core set of functions (e.g., “housekeeping” genes), it is the genes that vary between species that are often of most interest. For our framework to be a useful tool for reconstructing microbial genomes from metagenomic samples, it must perform well on such highly variable (or taxa-specific) genes. To further examine the ability of our framework to correctly predict such genes, we again used our reference genomes-based synthetic community model. We specified the set of variable genes in two ways: Those that are present in only one of the reference genomes composing the community and are absent from other community members, or those that are generally present in only very few bacterial genomes. We found that for genes that are present in only one species in the community, reconstructed genomes are 92% accurate with a recall of 96% (see also Figure 4B). Similarly, focusing on the set of genes found in less than 20% of all bacterial genomes in the KEGG database, we found that reconstructed genomes are 91% accurate with a recall of 97%. These results highlight the strength of the metagenomic deconvolution framework and its ability to correctly identify variable, taxa-specific genes and detect differences between genomes.

# Reconstructing genomes from a 20-strain microbial community based on the HMP Mock Communities

Metagenomic samples derived from a 20-strain microbial community based on the HMP Mock Community B [14] (http://www.beiresources.org/Catalog/otherProducts/HM-276D.aspx) were simulated to evaluate genome reconstructions for more complex model microbial communities with sequencing and annotation errors. 20 communities were generated as linear combinations of the reference organisms present in HMP Mock Community B (Dataset S3). Complete strain genomes were obtained from the Integrated Microbial Genomes database [52]. Strains had 16S copy numbers ranging from 1 to 14 copies. Each community had species relative abundances assigned randomly between 0 and 1 (Dataset S3). Metagenomic samples were generated for each community by simulating 10M shotgun metagenomic sequencing reads with Metasim [41], using 80-base reads. In contrast to the 3 strain communities dataset, reads were not BLAST-annotated, but were rather labeled with the “known” annotation of each read (i.e., according to the gene from which it originated using the IMG KO annotations), and a synthetic annotation error was added at a rate derived from the 3-strain community dataset (as specified in Dataset S2). Specifically, reads for which an erroneous annotation was chosen were assigned with an incorrect KO from a random protein-coding gene. Reads from RNA were not mis-annotated with a protein-coding gene. The metagenomic deconvolution framework was then applied to reconstruct the genomic content of each strain, using strain abundances derived from the relative abundances of 16S genes in the community. Using a threshold of 0.05 of the average KO length, genomes were reconstructed with an accuracy of 87% and a recall of 80%. We find that genome reconstructions created using the metagenomic deconvolution framework were accurate even with the large error in 16S-based abundances. The magnification of the apparent taxonomic abundances due to 16S copy number variation resulted in shorter gene length predictions, but the use of a threshold to predict gene presences and absences corrected for this error. In contrast, the null model we discussed in the main text had an accuracy of 77% and a recall of 64% in reconstructing these genomes. Deconvolution-based genome reconstructions were significantly more accurate than such a convoluted null model regardless of the threshold used (P < 10^-324^, bootstrap; Figure S8).

# Deconvolving simple synthetic communities with species-specific gene lengths

In the main text, we applied the deconvolution framework to simple synthetic communities in which genes were assumed to have the same length across all species they were present in. To determine whether this assumption affected the accuracy of reconstructed genomes, we repeated our analysis with a new set of simulated communities in which genes varied in length from species to species. Specifically, each gene in each species was given a random length chosen from a normal distribution with 3% standard deviation around the gene’s standard length. We deconvolved these synthetic samples and reconstructed the genomic content of each simulated species as in the main text. We found no significant difference in the accuracy and recall of reconstructed genomes compared to the constant gene-length model (Figure S12).

# References

1. Baran Y, Halperin E (2012) Joint analysis of multiple metagenomic samples. PLoS Comput Biol 8: e1002373. doi:10.1371/journal.pcbi.1002373.

2. Karypis G, Han E, Kumar V (1999) Chameleon: Hierarchical clustering using dynamic modeling. Computer 32: 68–75.

3. Qin J, Li Y, Cai Z, Li S, Zhu J, et al. (2012) A metagenome-wide association study of gut microbiota in type 2 diabetes. Nature 490: 55–60. doi:10.1038/nature11450.

4. Van Dongen SM (2000) Graph clustering by flow simulation. PhD Thesis, University of Utrecht.

5. Karlsson FH, Tremaroli V, Nookaew I, Bergström G, Behre CJ, et al. (2013) Gut metagenome in European women with normal, impaired and diabetic glucose control. Nature: 4–10. doi:10.1038/nature12198.

6. Devarajan K (2008) Nonnegative matrix factorization: an analytical and interpretive tool in computational biology. PLoS Comput Biol 4: e1000029. doi:10.1371/journal.pcbi.1000029.

7. Jiang X, Langille MGI, Neches RY, Elliot M, Levin S a, et al. (2012) Functional biogeography of ocean microbes revealed through non-negative matrix factorization. PLoS One 7: e43866. doi:10.1371/journal.pone.0043866.

8. Jiang X, Weitz JS, Dushoff J (2012) A non-negative matrix factorization framework for identifying modular patterns in metagenomic profile data. J Math Biol 64: 697–711. doi:10.1007/s00285-011-0428-2.

9. Chen M (2010) K-medoids (http://www.mathworks.com/matlabcentra l/fileexchange/28898-k-medoids). MATLAB Central File Exchange.

10. Karypis G, Kumar V (1998) A fast and high quality multilevel scheme for partitioning irregular graphs. SIAM J Sci Comput 20: 359–392.

11. Li Y, Ngom A (2013) The non-negative matrix factorization toolbox for biological data mining. Source Code Biol Med 8: 10. doi:10.1186/1751-0473-8-10.

12. Tibshirani R (1996) Regression shrinkage and selection via the lasso. J R Stat Soc Series B Stat Methodol 58: 267–288.

13. Qin J, Li R, Raes J, Arumugam M, Burgdorf KS, et al. (2010) A human gut microbial gene catalogue established by metagenomic sequencing. Nature 464: 59–65. doi:10.1038/nature08821.

14. Methé BA, Nelson KE, Pop M, Creasy HH, Giglio MG, et al. (2012) A framework for human microbiome research. Nature 486: 215–221. doi:10.1038/nature11209.
